# Assessment of the effectiveness of the EUROFORGEN NAME and Precision ID Ancestry panel markers for ancestry investigations

**DOI:** 10.1038/s41598-021-97654-0

**Published:** 2021-09-20

**Authors:** D. Truelsen, T. Tvedebrink, H. S. Mogensen, M. S. Farzad, M. A. Shan, N. Morling, V. Pereira, C. Børsting

**Affiliations:** 1 Section of Forensic Genetics, Department of Forensic Medicine, Faculty of Health and Medical Sciences, University of Copenhagen, 2100 Copenhagen, Denmark; 2 Department of Mathematical Sciences, Aalborg University, 9220 Aalborg, Denmark

**Keywords:** Genomics, Sequencing, Genetics, Genetic markers, Genotype, Population genetics, Sequencing

## Abstract

The EUROFORGEN NAME panel is a regional ancestry panel designed to differentiate individuals from the Middle East, North Africa, and Europe. The first version of the panel was developed for the MassARRAY system and included 111 SNPs. Here, a custom AmpliSeq EUROFORGEN NAME panel with 102 of the original 111 loci was used to sequence 1098 individuals from 14 populations from Europe, the Middle East, North Africa, North-East Africa, and South-Central Asia. These samples were also sequenced with a global ancestry panel, the Precision ID Ancestry Panel. The GenoGeographer software was used to assign the AIM profiles to reference populations and calculate the weight of the evidence as likelihood ratios. The combination of the EUROFORGEN NAME and Precision ID Ancestry panels led to fewer ambiguous assignments, especially for individuals from the Middle East and South-Central Asia. The likelihood ratios showed that North African individuals could be separated from European and Middle Eastern individuals using the Precision ID Ancestry Panel. The separation improved with the addition of the EUROFORGEN NAME panel. The analyses also showed that the separation of Middle Eastern populations from European and South-Central Asian populations was challenging even when both panels were applied.

## Introduction

The identification of perpetrators of crimes by DNA investigations may be hindered by the absence of a reference sample from the offenders or database hits. In such cases, there is a need for additional DNA analyses that can lead the investigation in a specific direction^1,2^. Analysis of ancestry informative markers (AIMs) may be used to infer the biogeographic ancestry of the individual that left the trace sample at the crime scene^3^. Sets of AIMs can be combined to target different geographical regions on large or small geographical scales. The resolution of ancestry at the continental level, e.g. Africa, Europe, East Asia, and the Americas has been achieved using commercially available panels including the Precision ID Ancestry Panel and the ForenSeq DNA Signature Prep Kit^4–9^. However, such panels have limited success with population assignment of individuals with admixed ancestries and may not be able to differentiate individuals from regions that have experienced multiple population migration events.

From a population genetic perspective, the Middle East is a particularly interesting region. The Middle East (covering Turkey in the West to Afghanistan in the East) connects Africa, South-Central Asia, and Europe and has a history of many human migration events^10^. Within the last 5000 years, many different powerful empires have dominated the region, fighting each other for political control and subsequently declining in power after relatively short periods of reign^11^. As a consequence, the borders in the Middle East changed and parts of the populations migrated each time a new empire gained power^12–14^. Migration of individuals to and from the surrounding regions further reduced the level of genetic divergence between the Middle Eastern, European, and South-Central Asian populations ^15^. The current political borders between the countries in the Middle East were agreed between the United Kingdom and France in 1916^11,16^, and they do not reflect the genetics of the Middle East populations^12,13,17,18^. Furthermore, the Middle East is situated in the centre of an allele frequency gradient from North-Western Europe to East Asia^15,19^. This makes it particularly difficult to differentiate individuals from the Middle East, Europe, and South-Central Asia^15^.

The EUROFORGEN North Africa and Middle East (NAME) panel is a regional ancestry panel for ancestry inference of individuals from the Middle East and North Africa^20^. It includes 111 AIMs and was designed to improve the population assignment of individuals from the Middle East, North Africa, and Europe. The AIMs were amplified with four PCR multiplexes and analysed using the MassARRAY System. Each multiplex required 2.5–10 ng DNA, which is more than what is recovered from the majority of crime scene samples. Therefore, a custom AmpliSeq assay was developed for the Ion S5 platform^21^. Of the 111 loci in the EUROFORGEN NAME panel, 102 loci were successfully amplified and sequenced with as little as 0.5–1 ng DNA.

In this work, we typed 1098 individuals from 14 populations from the Middle East, North Africa, North-East Africa, South-Central Asia, and Europe for 265 AIMs using the AmpliSeq EUROFORGEN NAME panel and the Precision ID Ancestry Panel (Thermo Fisher Scientific)^6^. We used the method developed for the GenoGeographer software^22^ to assess whether the use of the combination of the EUROFORGEN NAME and Precision ID Ancestry Panel markers would improve the correctness of the assignment of individuals to their population of origin. It was further investigated if the combination of the panels increased the weight of the evidence of the assignment of the population of origin.

## Results

Of the 1098 individuals analysed, 336 were typed with the MassARRAY EUROFORGEN NAME assay^20^, and 762 were typed with the custom AmpliSeq EUROFORGEN NAME panel^21^. Only the 102 loci that were included in both assays were used for the population genetic and ancestry analyses below. The information concerning the physical position and rs-numbers of the loci included in the AmpliSeq design is shown in Supplementary Table S1. All samples were also typed for the 165 AIMs of the Precision ID Ancestry Panel^6,21^ and in this work. Two AIMs, rs12913832 and rs4833103, were present in both the EUROFORGEN NAME panel and the Precision ID Ancestry Panel. These two AIMs performed best in the EUROFORGEN NAME panel, and the results from the Precision ID Ancestry Panel were not used. Of the 1098 individuals, 28 individuals had no genotype calls in more than 10% of the loci. The data of these individuals were excluded from further analysis. Data for the remaining 1070 individuals were used for the downstream analyses.

The data obtained with the EUROFORGEN NAME and Precision ID Ancestry panels were tested separately for Hardy–Weinberg equilibrium (HWE). For the EUROFORGEN NAME panel, the data of the AIM rs7873963 was in Hardy–Weinberg disequilibrium in five populations (P_cor_ = 4.9E-04). There was an excess of homozygotes of the T allele, which was caused by a deletion downstream of the locus that was associated with the C allele. Only samples typed with the MassARRAY assay were affected by the deletion; the locus was in HWE in the populations typed with the AmpliSeq EUROFORGEN NAME panel. The locus, which was also in linkage disequilibrium (LD) with another locus (see below), was excluded from further population genetic analysis.

The HWE was also assessed for the markers present in the Precision ID Ancestry panel. After Bonferroni correction, the AIM rs310644 was in Hardy–Weinberg disequilibrium in the Pakistani and Portuguese populations (P_cor_ = 3.07E−4). Among Portuguese individuals, 74 had the TT genotype, two had the CC genotype, while no heterozygote individual was observed. Among Pakistani individuals (N = 72), 43 individuals had the TT genotype, 13 the CC genotype, and 16 the CT genotype.

Linkage disequilibrium (LD) analysis was performed on the combined dataset including 265 AIMs with 34,980 pairs of loci. Besides LD most likely due to physical linkage, LD between alleles at different chromosomes was also observed. Supplementary Tables S4 and S5 show the pairs of loci that were in statistically significant LD in the different populations. Several loci in the EUROFORGEN NAME panel showed statistically significant LD. The *HaploView* software was used to evaluate if these loci could belong to haplotype blocks. The analysis showed that two groups of markers on chromosome 4 (rs4975193—rs1757928—rs337277—rs1699387, and rs17616434—rs4833103), one group on chromosome 7 (rs9649356—rs1227171), one group on chromosome 10 (rs2031581—rs2765650), and one group on chromosome 12 (rs10862511—rs10506882) seemed to form haplotype blocks. The loci rs1406045 (typed with the EUROFORGEN NAME panel) and rs4463276 (typed with the Precision ID Ancestry Panel) on chromosome 6 as well as rs621341, typed with the EUROFORGEN NAME panel, and rs6754311, typed with the Precision ID Ancestry Panel on chromosome 2 were in linkage disequilibrium (Supplementary Table S4). To ensure marker independence, one locus in each pairwise comparison was eliminated for the population genetic analyses. The performance of the loci in terms of heterozygote balance, locus balance, noise level, and the number of genotype drop-outs was evaluated and for each pair, the locus with the best performance was retained. If the loci performed equally well, preference was given to the locus with the shortest read length (Supplementary Table S6). After evaluating the LD, the final numbers of loci for further genetic analysis were 72 for the EUROFORGEN NAME panel and 161 for the Precision ID Ancestry Panel. The combined dataset included 233 SNP markers.

### Genetic structure

**Figure 1:**
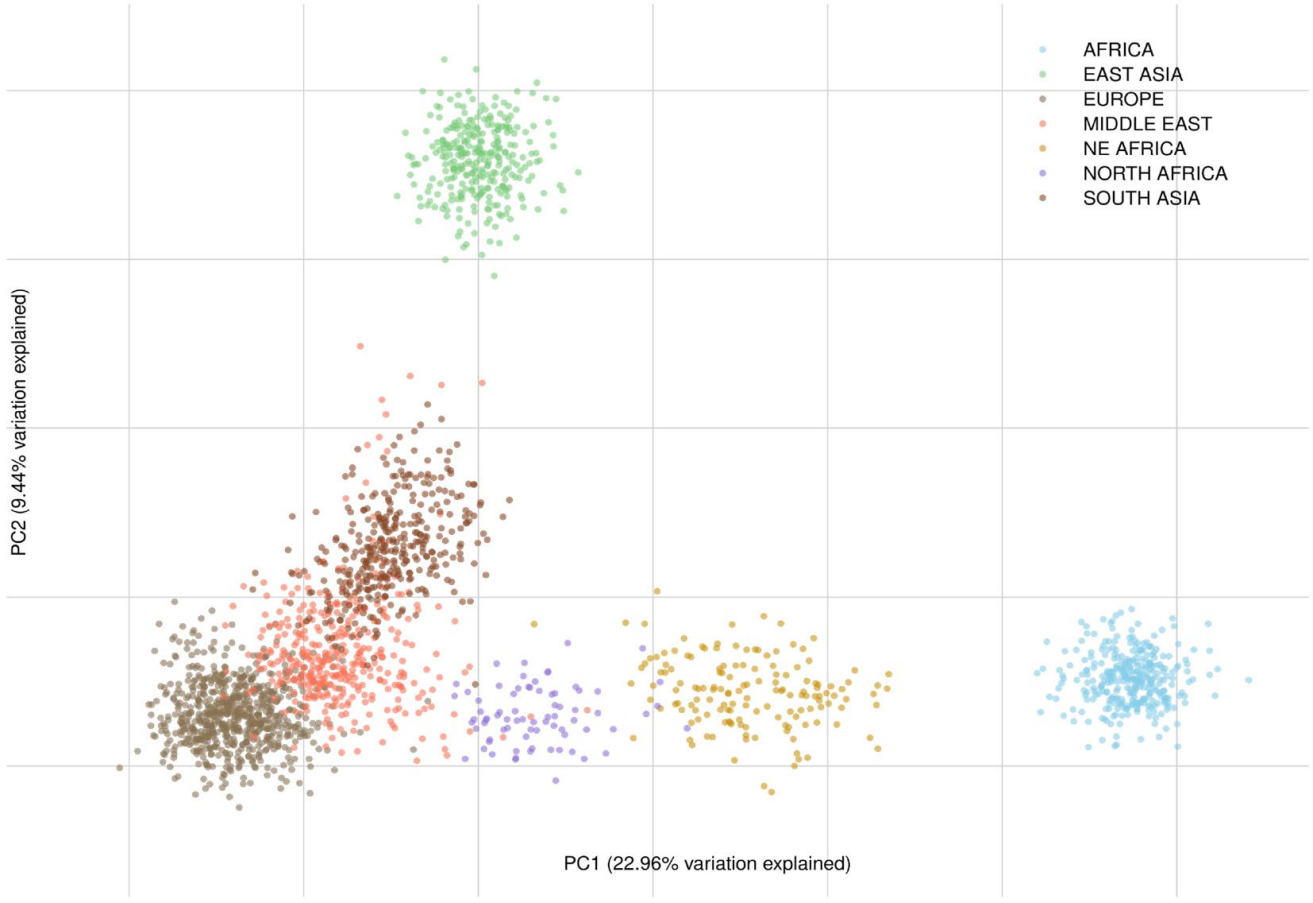
PCA plot of the results obtained with the combined dataset of 233 AIMs included in the EUROFORGEN NAME panel and the Precision ID Ancestry Panel. The PCA were performed using a custom script written in R v. 3.5.0 using the ‘adegenet’ v. 2.1.2 and the ‘ade4’ v. 1.7-15 R packages^43,44^.

The population variation of reference groups from Sub-Saharan Africa (N = 606), Europe (N = 604), the Middle East (N = 134), South-Central Asia (N = 689), and East Asia (N = 504) was analysed. Figure 1 shows a PCA plot of the combined data set with 233 AIMs. PCAs where each population is highlighted can be found in the supplementary materials (Supplementary Figures S1–S14). The Sub-Saharan African, European, South Asian, and East Asian individuals were separated from each other by PC1 and PC2. The Middle Eastern individuals was located between the South Asian and the European individuals with a small overlap with the European individuals. The North African individuals were situated between the Sub-Saharan African and the Middle Eastern individuals, while the NE African individuals were found between the North African and Sub-Saharan African individuals. Supplementary Figure S15 shows a similar analysis based on the 72 EUROFORGEN NAME markers only. PCA analyses showed that the Middle Eastern individuals had a larger overlap with the Southern European populations from Greece and Albania than with the Danish individuals (Supplementary Figures S2, S3, and S5). There was a substantial overlap between the Middle Eastern and South-Central Asian populations mainly consisting of individuals from Afghanistan.

**Figure 2:**
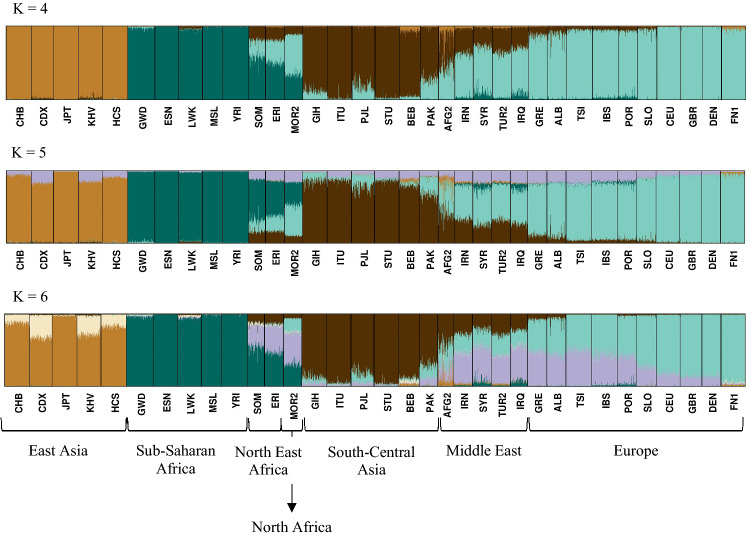
Diagram of the STRUCTURE analysis with runs of K = 4 to 6 of the combined dataset of 233 AIMs. The reference data are from the 1000 Genomes Project. Population abbreviations are the same as those described in Supplementary Materials Table S2. The membership proportions were plotted using CLUMPP v.1.1.222^48^ and Distruct v. 1.1.23^49^.

To evaluate the genetic structure of the populations, STRUCTURE analyses were performed using K = 3 to K = 7. Figure 2 shows the results for K = 4 to K = 6 for the 233 loci in the combined data set. The most likely number of clusters was K = 4 corresponding to the Sub-Saharan, East Asian, South-Central Asian, and European populations. Co-ancestry contribution from Sub-Saharan, European, and South-Central Asian populations was observed among individuals from North-East Africa and North Africa, whereas the Middle Eastern individuals shared cluster memberships with primarily the European populations and, to a smaller degree, South-Central Asians. With K = 6, an additional component was observed for the Middle Eastern, North-East African, and the European individuals. For the Middle Eastern individuals, the component differed from those of the North-East African and North African populations mainly due to the Sub-Saharan contribution to the latter populations, and it differed from the clusters of the European populations due to the South-Central Asian contribution to the cluster. Some variation within the European cluster was also observed at K = 6. South Europeans shared more cluster membership with the Middle Eastern, North-East African, and North African populations than the North Europeans. The STRUCTURE analysis performed with EUROFORGEN NAME markers only showed a similar pattern (Supplementary Figure S16).

### Population assignment based on z-score and LR

Based on the STRUCTURE and PCA results, the 14 populations typed in this work were grouped into five meta-populations: (1) a European meta-population including individuals from Albania, Denmark, Greece, Portugal, and Slovenia, (2) a Middle Eastern meta-population including individuals from Afghanistan, Iran, Iraq, Syria, and Turkey, (3) a North-East African meta-population including individuals from Eritrea and Somalia, (4) a North-African meta-population including individuals from Morocco, and (5) a South-Central Asian meta-population including individuals from Pakistan.

A z-score test was performed for each of the 1070 individuals using the GenoGeographer software and the cross-validation method^22,23^. This was done for the EUROFORGEN NAME panel (72 loci), the Precision ID Ancestry Panel (161 loci), and the combined dataset (233 loci). The AIM profiles were tested against both the individual’s meta-population of origin and the four other meta-populations. Table 1 shows the results of the z-score tests. The results of the test of each AIM profile against each meta-population with the three sets of AIMs were categorised as either “Accepted”, “Ambiguous”, or “Rejected” (Fig. 4).

**Table 1:** Effectiveness of the ancestry estimation with the Precision ID Ancestry Panel, the EUROFORGEN NAME panel, and the combined panel.

			Accepted AIM profile*			Ambiguous AIM profile**			Rejected AIM profile***		
Population of origin	Meta-population size	Population against which the individual was tested	Precision ID (%)	NAME (%)	All (%)	Precision ID (%)	NAME (%)	All (%)	Precision ID (%)	NAME (%)	All (%)
Europe	398	Europe	81.7	63.8	82.9	12.1	29.9	10.6	6.3	6.3	6.5
		Middle East	3.5	6.8	3.3	10.6	16.1	8.3	85.9	77.1	88.4
		North Africa	0.3	0.3	0.5	0.5	2.5	0.0	99.2	97.2	99.5
		North-East Africa	0.0	0.0	0.0	0.0	0.3	0.0	100.0	99.7	100.0
		South-Central Asia	0.5	3.8	0.8	3.3	20.6	2.8	96.2	75.6	96.5
Middle East	371	Europe	3.3	4.3	1.1	15.6	13.5	8.6	80.9	82.2	90.3
		Middle East	38.5	25.9	49.9	47.4	63.3	36.7	14.0	10.8	13.5
		North Africa	1.1	2.7	1.3	6.2	7.0	4.6	92.7	90.3	94.1
		North-East Africa	0.0	0.0	0.0	0.0	2.2	0.0	100.0	97.8	100.0
		South-Central Asia	4.3	11.3	5.7	29.4	50.1	26.1	66.3	38.5	68.2
North Africa	75	Europe	0.0	0.0	0.0	0.0	0.0	0.0	100.0	100.0	100.0
		Middle East	1.3	0.0	0.0	1.3	2.7	0.0	97.3	97.3	100.0
		North Africa	90.7	70.7	85.3	5.3	20.0	2.7	4.0	9.3	12.0
		North-East Africa	0.0	0.0	0.0	2.7	17.3	2.7	97.3	82.7	97.3
		South-Central Asia	0.0	0.0	0.0	1.3	0.0	0.0	98.7	100.0	100.0
North-East Africa	149	Europe	0.0	0.0	0.0	0.0	0.0	0.0	100.0	100.0	100.0
		Middle East	0.0	0.0	0.0	0.0	0.0	0.0	100.0	100.0	100.0
		North Africa	0.0	3.4	0.0	4.0	20.1	2.7	96.0	76.5	97.3
		North-East Africa	87.9	69.8	87.2	4.0	20.1	2.7	8.1	10.1	10.1
		South-Central Asia	0.0	0.0	0.0	0.0	0.0	0.0	100.0	100.0	100.0
South-Central Asia	77	Europe	0.0	1.3	0.0	0.0	11.7	1.3	100.0	87.0	98.7
		Middle East	2.6	2.6	1.3	20.8	45.5	14.3	76.6	51.9	84.4
		North Africa	0.0	1.3	0.0	0.0	1.3	0.0	100.0	97.4	100.0
		North-East Africa	0.0	0.0	0.0	0.0	0.0	0.0	100.0	100.0	100.0
		South-Central Asia	68.8	46.8	77.9	20.8	50.6	14.3	10.4	2.6	7.8

*Accepted: The AIM profi1e was (1) accepted in only one meta-population (z-score ≤ 1.64; *P* ≥ 0.05) or (2) accepted in more than one meta-population (z-score ≤ 1.64; *P* ≥ 0.05) and the likelihood of the AIM profile belonging to the population was statistically significantly higher than those of all other likelihoods (*P* < 0.05).

**Ambiguous: The AIM profile was (1) accepted in more than one meta-population (z-scores ≤ 1.64; *P* ≥ 0.05) and (2) the population likelihoods were not statistically significantly different from each other (z-scores ≤ 1.64; *P* ≥ 0.05).

***Rejected: The AIM profile was not accepted in any meta-population (z-score > 1.64; *P* < 0.05).

Irrespectively of the origin of the sample, the number of AIM profiles categorised as “Ambiguous” was lower with the combined set of markers than with the Precision ID Ancestry Panel. The reduction in the number of ambiguous profiles was most pronounced for individuals from the Middle East and South-Central Asia (Table 1). In both cases, the population assignments primarily changed from “Ambiguous” to “Accepted”. For example, 47.4% of the Middle Eastern individuals were classified as “Ambiguous” with the Precision ID Ancestry Panel, while only 36.7% were classified as “Ambiguous” with the combined panel. The percentage of Middle Eastern individuals in the “Accepted” category increased from 38.5% with the Precision ID Ancestry Panel to 49.9% with the combined panel. Furthermore, fewer Middle Eastern individuals, categorised as “Accepted” or “Ambiguous”, likely belonged to the European meta-population based on the genotypes generated with the combined panel (1.1% and 8.6%, respectively) compared to the genotypes generated with the Precision ID Ancestry Panel (3.3% and 15.6%, respectively).

For the North African and the North-East African meta-populations, the number of profiles assigned to the ‘Rejected’ category increased when the combined panel was used. Regarding North African individuals, four profiles classified as ‘Accepted’ and two profiles classified as ‘Ambiguous’ with the Precision ID Ancestry panel were assigned as ‘Rejected’ with the combined panel. For the North-East African individuals, three profiles (one defined as ‘Accepted’ and two as ‘Ambiguous’) were classified as ‘Rejected’ when the combined panel was used. These AIM profiles were outliers in all reference populations (z-scores > 1.64; *P* < 0.05) with the combined panel.

**Figure 3:**
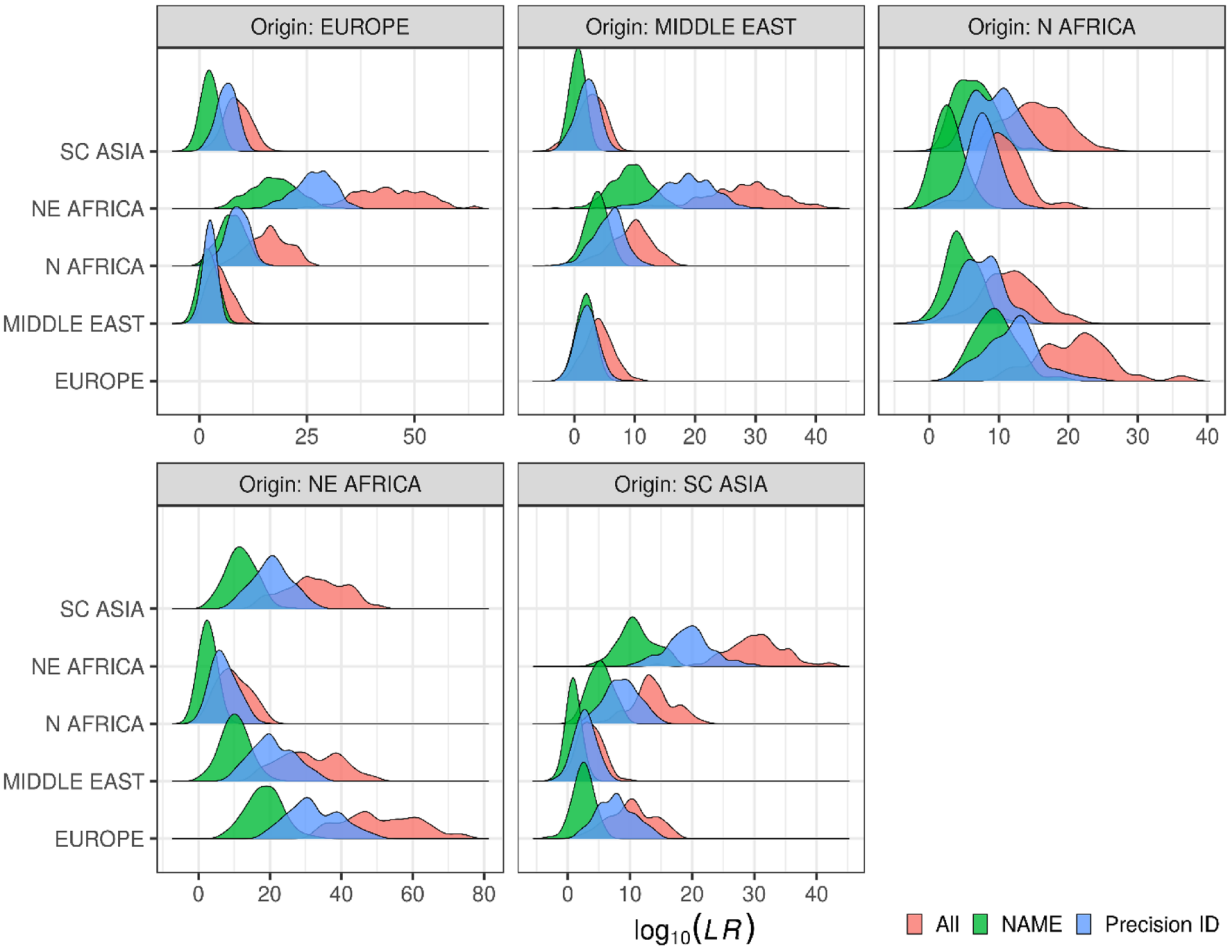
Distributions of log LRs for the individuals with z-score ≤ 1.64 (*P* ≥ 0.05) for the population of origin (listed in the plot headings). The colours refer to the panels used. The curves are based on smoothed kernel density estimates for the 1070 individuals. The hypothesized meta-population in the numerator of the LRs is given by the heading of each plot, while the hypothesized meta-population in the denominator is indicated to the left of the ordinate. R v. 3.5.0 and the *‘ggplot2’* v. 3.2.1 R package (https://ggplot2.tidyverse.org/index.html) was used to visualise the LRs.

Figure 3 shows the distribution of the log LRs for all individuals with z-scores ≤ 1.64 (*P* ≥ 0.05) for their populations of origin. Overall, the combined panel (red distribution in Fig. 3) led to an increase in LRs compared to those of the two panels separately. The increase in LR for the combined panel was greatest when the AIM profiles of individuals from North Africa and North-East Africa were compared with those from individuals from Europe, the Middle East, and South-Central Asia, while it was smallest when the AIM profiles of individuals from (1) Europe and the Middle East and (2) the Middle East and South-Central Asia were compared.

**Figure 4:**
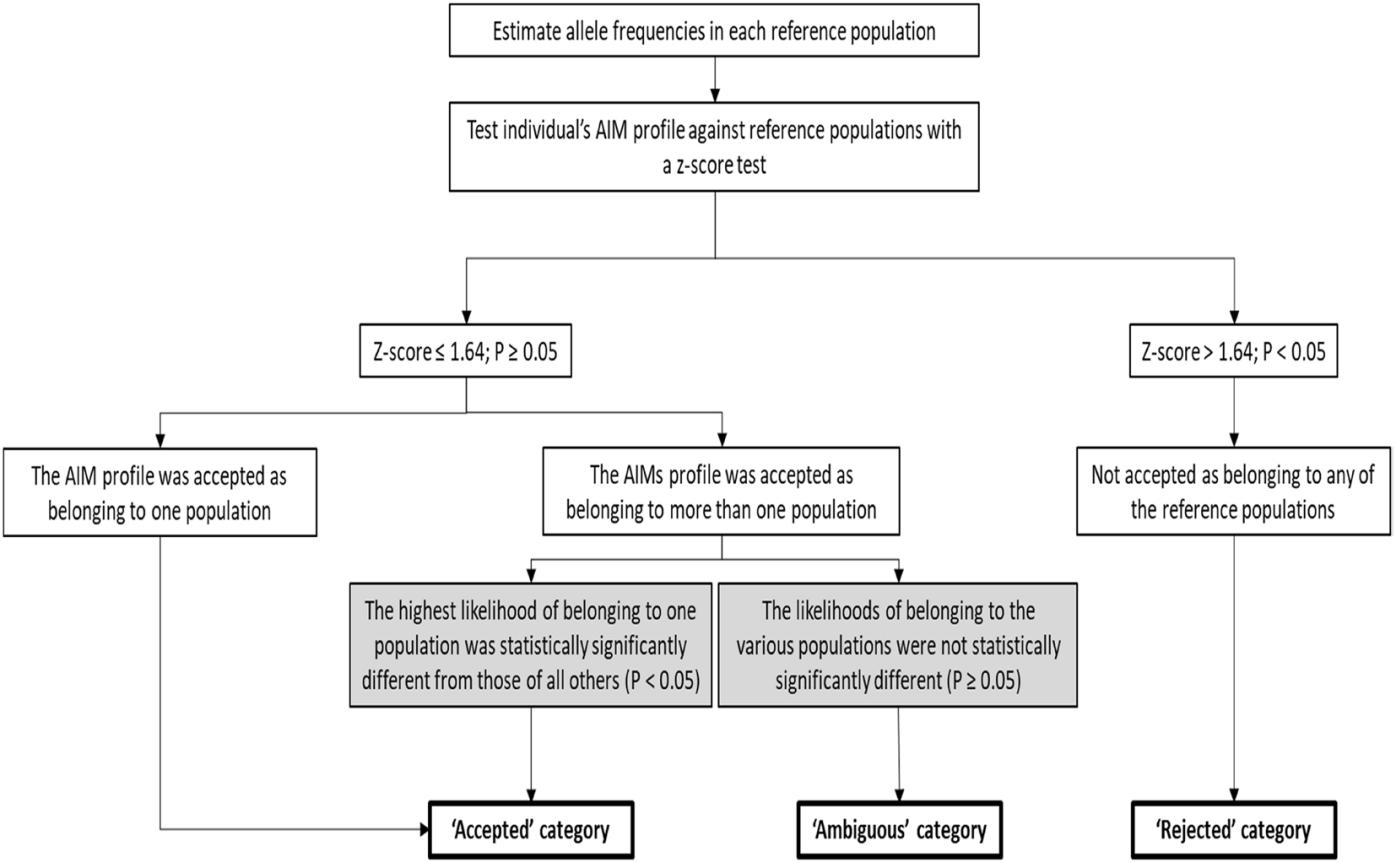
Diagrammatic presentation of the decisions for classification of the results of investigations of ancestry with AIMs.

## Discussion

With commercial Massively Parallel Sequencing ancestry panels such as the Precision ID Ancestry Panel and the ForenSeq DNA Signature Prep Kit, a continental differentiation is now possible^6,7,24^. The panels work well as global ancestry panels; the purpose of which is to explore whether a DNA sample from an unidentified individual could originate from any of the major geographical regions of e.g. Sub-Saharan Africa, Europe, East Asia, and the Americas^6,7,25–30^. The interest is now shifting towards regional ancestry panels and their practical use for ancestry inference in forensic casework. From a Danish perspective, the ability to separate individuals of European descent from those of Middle Eastern, North African, and North-East African descent is particularly interesting due to the recent immigration events to Denmark from these regions. The effectiveness of separating individuals from the above-mentioned populations with a global ancestry panel is limited because the AIMs in these panels were selected to separate individuals from the major, continental populations^31–33^. Therefore, the use of regional ancestry panels for the separation of individuals on a finer geographical scale is relevant.

The design of custom panels with online design tools is relatively straightforward^21,34^. Thus, the number of custom-made panels for the assignment of individuals to specific population groups will most likely increase in the future. The EUROFORGEN NAME panel was designed for the identification of individuals of North African and Middle Eastern ancestries. The original MassARRAY version of the EUROFORGEN NAME panel included 111 loci amplified by four separate multiplex PCRs. In contrast, the AmpliSeq panel tested in this work amplified 102 loci in one multiplex PCR^21^. In the present work, it was assessed if the EUROFORGEN NAME AmpliSeq panel would be valuable as a supplement, i.e. a regional ancestry panel, to the Precision ID Ancestry Panel for the assessment of the ancestry of individuals most likely from either Europe, the Middle East, North Africa, North-East Africa, or South-Central Asia. Some loci of the EUROFORGEN NAME panel and/or the Precision ID Ancestry Panel were shown to be in linkage disequilibrium with each other. Therefore, 32 markers were excluded for further population genetic and ancestry analysis to maintain the a priori assumption that the genetic markers used for the analysis were independent and in HWE. Together, the Precision ID Ancestry Panel and the EUROFORGEN NAME panel include 233 different genetic SNP markers. The PCA plots and the STRUCTURE analyses (Figs. 1 and 2 as well as Supplementary Figures S1–S16) confirmed that the combined panel increased the separation of the European and Middle Eastern population groups compared to that of the Precision ID Ancestry Panel alone. Although PCA and STRUCTURE analyses provide easy ways to visualise data clustering, these methods are empirical and not adequate for ancestry inference in forensic genetics, as they cannot calculate the statistical weight of the results.

For forensic genetic population assignment, sufficient population reference data and their geographical distribution play a major role^4,7,23^. The data from the reference populations are used to estimate the likelihood of an AIM profile. The population with the highest likelihood is often reported as the population of origin of that individual^4,35^. However, if the true population of origin is not present in the population reference data, this assignment is incorrect and misleading^23^. The method implemented in the GenoGeographer software^22^ includes a statistical z-score test that evaluates if an appropriate reference population is present among the reference data. Calculation of the statistical weight should not be performed if the AIM profile does not belong to any of the reference populations^22^. Here, we used GenoGeographer to evaluate if the combination of a global and a regional ancestry panel increased the differentiation power compared to each of the panels separately. The combination of panels affected the rates of “Accepted”, “Rejected”, and “Ambiguous” ancestry profiles in the different meta-populations and increased the LRs. The positive effect of using both panels was strongest for Middle Eastern and South-Central Asian individuals. When the combined panel was used instead of the individual panels, the number of “Accepted” ancestry profiles increased and the number of “Ambiguous” results decreased. Additionally, the rate of “Rejected” ancestry profiles increased when an individual was compared to an incorrect meta-population. For the North African meta-population (Moroccans) and the North-East African meta-population (Eritreans and Somalis), 15 AIM profiles were “Rejected” from their true meta-populations of origin with the Precision ID Ancestry Panel, while 24 AIM profiles were “Rejected” with the combined panel. These individuals may belong to populations that were not included among the reference populations, or they may have mixed ancestries. In both cases, investigations of neighbouring populations, including Sub-Saharan populations, would have been relevant.

LRs were calculated for individuals that were assigned to their assumed population of origin (“Accepted” and “Ambiguous” categories) (Fig. 3). The use of the combined panel increased the LRs for all comparisons. The reporting of the results of AIM testing in forensic genetics is based on the likelihood ratio principle that is recommended by the International Society for Forensic Genetics^36,37^. In the assessment of ancestry, however, there is a logic challenge in situations in which both likelihoods in the LR are based on hypotheses that are nonsense. Methods based on the likelihood principle are—on their own—not well suited for the evaluation of the plausibility of the hypotheses of the ancestry of an individual with a particular AIM profile. To avoid calculating LRs based on two nonsense-hypotheses—which would make such an LR of no use—we introduced a prior-test of the probabilities of the hypotheses based on the z-score test. If both hypotheses are rejected, no further calculation of LR is performed. In practical forensic genetic work, this means that LRs are only calculated if the AIM profile of the individual is accepted in at least one of the reference populations and the LR will give meaningful information. If an AIM profile is accepted to belong to only one population, it is still relevant to calculate the LRs based on comparison with all other relevant populations to assess the strength of the evidence. If an AIM profile is accepted to belong to two or more populations, the various LRs based on comparisons between the relevant populations can be calculated. If any LR is statistically significantly higher than any other, this will be strong evidence in favour of the individual belonging to the population resulting in the highest likelihood. Again, the strength of the evidence is given by the LR. It must, however, be taken into consideration that the pure fact that an AIM profile based on a z-score value ≤ 1.64 (*P* > 0.05) is accepted to belong to one—and only one—tested population does not prove that the individual belongs to that particular population. The individual may instead belong to another population that is genetically close to the tested population, or the individual may be of admixed ancestry with sufficient contribution from the tested population to allow the admixed AIM profile to be accepted into that population. The individual could also belong to a population that is not present in the reference database. Thus, in a case with a question to which of two populations an individual belongs, the first step would be to perform a z-test. If the AIM profile can belong to any of the tested populations, the LR is calculated as the weight of the evidence. If the results of the z-test indicate that the AIM profile for practical purposes cannot belong to any of the tested populations, no further calculation is performed, and the conclusion is that the contributor of the AIM profile does not belong to any of the proposed populations.

The analyses performed in this work demonstrated that the addition of the loci in the EUROFORGEN NAME panel to those of the Precision ID Ancestry Panel improved the efficiency of the population assignment of individuals from Europe, the Middle East, North Africa, North-East Africa, and South-Central Asia. However, when comparing the performance of the panels separately, the Precision ID Ancestry Panel had higher inclusion and rejection rates than the EUROFORGEN NAME panel, which was designed as a regional ancestry panel and not as a stand-alone panel.

## Materials and methods

### Samples, DNA extraction, and quantification

A total of 1098 samples were selected from the Biobank of the Department of Forensic Medicine, University of Copenhagen, Denmark (Danish agency for data protection, ref. no. 2002-54-1080). All samples were anonymized. According to the Danish Act on Research Ethics Review of Health Research Projects, the work did not require approval by the Ethics Committee. The authors confirm that all methods were performed in accordance with the relevant guidelines and regulations. A total of 336 individuals from Albania, Denmark, Greece, Iraq, Slovenia, Somalia, and Turkey were previously typed with the MassARRAY EUROFORGEN NAME assay^20^. To reach a minimum of 75 individuals per population^23^, samples from 220 individuals from these populations were typed with the AmpliSeq EUROFORGEN NAME panel (Supplementary Table S1)^21^. Furthermore, 542 individuals from Afghanistan, Eritrea, Iran, Morocco, Pakistan, Portugal, and Syria were typed with the AmpliSeq EUROFORGEN NAME panel. All samples were also typed with the Precision ID Ancestry Panel (Thermo Fisher Scientific), either in this or previous works^6,23,31^.

DNA was extracted from either blood samples using the QIAamp DNA Blood Mini Kit (Qiagen, Hilden, Germany) or from blood or buccal swabs on FTA cards (Whatman Inc., Clifton, NJ) with the BioRobot EZ1 Workstation (Qiagen, Hilden, Germany) and the QIAamp DNA Investigator Kit (Qiagen, Hilden, Germany).

DNA extracts were quantified with the Qubit dsDNA High Sensitivity (HS) Assay Kit (Thermo Fisher Scientific) and a Qubit 3.0 Fluorometer (Thermo Fisher Scientific).

### Library preparation and DNA sequencing

DNA libraries were built using the AmpliSeq EUROFORGEN NAME panel and the Precision ID Ancestry Panel (Thermo Fisher Scientific) and the Ion AmpliSeq Library Kit. 2.0 (Thermo Fisher Scientific) according to the manufacturers’ recommendations, except for using 25 PCR cycles^6^ and preparing the libraries using half volume of reagents. The DNA input ranged from 0.3 to 1 ng. The DNA libraries were purified with Agencourt AMPure XP magnetic beads (Beckman Coulter Inc., CA, USA) with a Biomek 3000 Laboratory Automation Workstation (Beckman Coulter Inc., CA, USA)^38,39^. The barcoded libraries were quantified using a Qubit Fluorometer and the Qubit dsDNA HS Assay Kit (Thermo Fisher Scientific). The DNA libraries were subsequently pooled in equimolar concentrations (28–35 pM) using a Biomek 3000 Laboratory Automation Workstation (Beckman Coulter Inc., CA, USA)^38,39^. The template preparation (emulsion PCR, enrichment of Ion Sphere particles, and chip loading) was performed on an Ion Chef instrument (Thermo Fisher Scientific) using the Ion S5 Precision ID Chef Kit (Thermo Fisher Scientific). Approximately 80 libraries were loaded per Ion 530 chip. A negative control was included per chip. Sequencing was performed with an Ion S5 (Thermo Fisher Scientific) using the Ion S5 Precision ID Sequencing Kit (Thermo Fisher Scientific).

### Data analysis

Sequence analysis was performed with data from BAM-files using the HID-SNP Genotyper v.5.2.2 plug-in on a Torrent Suite server. Target and hotspot BED-files were provided to specify the loci of interest in the human hg19 reference genome. The following default settings were used for the data analysis: Minimum allele frequency = 0.1, minimum coverage = 6, minimum coverage of either strand = 0, and maximum strand bias = 1. The plug-in generated CSV-files that were used for the downstream analysis. The Precision ID Ancestry Panel and AmpliSeq EUROFORGEN NAME DNA libraries were analysed separately as the datasets required different target and hotspot BED-files. To evaluate the quality of the data, the heterozygote balance (Hb) and noise level were calculated. The Hb was calculated as the number of reads for one nucleotide divided by the number of reads for the other nucleotide in the called genotype in the order A, C, G, and T. Noise was calculated as the number of reads that were different from the called genotype divided by the total number of reads. Two sets of genotype acceptance criteria were applied: (1) a minimum read depth ≥ 45 reads and 0.3 ≤ heterozygote balance ≤ 3.0; and (2) for loci with a read depth of 20 to 44, 0.7 ≤ heterozygote balance ≤ 1.3 and no noise. Genotypes that did not fulfil these criteria were named NN, i.e. no data.

Tests for Hardy–Weinberg equilibrium (HWE) of alleles were performed with the Arlequin v.3.5.2.2 software^40^ using 1,000,000 Markov chain steps. Pairwise tests for linkage disequilibrium (LD) between alleles was tested with Arlequin v.3.5.2.2 using an exact test. Only individuals with full profiles (no missing data) were used for the LD analysis. Since multiple tests were performed, the *P* values of analyses of HWE and LD were adjusted with the method of Bonferroni^41^ and indicated as P_cor_. Data from loci that showed statistically significant LD were further investigated using the HaploView v. 4.2 software to assess whether the loci were included in haplotype blocks using the default model^42^.

### Genetic structure

PCA and STRUCTURE analyses were performed with different reference data according to the availability of information concerning the markers. For the EUROFORGEN NAME markers, reference data included samples from the 1000 Genomes Project and HGDP-CEPH^20^ (Supplementary Table S2). For the combined dataset of EUROFORGEN NAME and Precision ID Ancestry Panels (referred to as ‘Combined’ in the following sections), PCA and STRUCTURE analyses were carried out using the reference data from the 1000 Genomes Project (Supplementary Table S3).

The principal component analyses (PCA) were performed using a custom-designed script written in R v. 3.5.0 using the ‘*adegenet*’ v. 2.1.2 and the ‘*ade4*’ v. 1.7-15 R packages^43,44^. The ancestral component of each individual was assessed using the software STRUCTURE v.2.3.4^45,46^. The clustering analysis was carried out using 100,000 steps for burn-in followed by 100,000 MCMC steps. Three to seven clusters (K) were explored. For each K, 10 iterations were performed for the EUROFORGEN NAME panel, and three for the combined panel. We used the admixed model with correlated allele frequencies. Population information was used for reference populations to help cluster formation (POPFLAG = 1). The optimal K was evaluated using STRUCTURE HARVESTER^47^. The membership proportions were plotted using CLUMPP v.1.1.222^48^ and Distruct v. 1.1.23^49^. Results from the PCA and the STRUCTURE analyses were used to group the 14 populations into meta-populations.

### GenoGeographer analysis

Three sets of data (the Precision ID Ancestry Panel, the EUROFORGEN NAME panel, and the combined panels) were compared to each other using the GenoGeographer software^22,23^. Using the z-score test, GenoGeographer first tested if the investigated AIM profile could be grouped with any of the reference populations in the database. The test included an estimation of the variance of the allele frequencies in the reference populations^50^. If the *P *value of the AIM profile belonging to a reference population was < 0.05 (z-score > 1.64), GenoGeographer rejected the hypothesis that the AIM profile belonged to that particular reference population (Fig. 4). For each AIM profile and marker panel, the z-score of the AIM profile was computed using cross-validation (out-of-sample) procedure. Here, the investigated AIM profile was compared with the reference AIM profiles by leaving out the investigated AIM profile. The z-scores were computed for all individuals against all meta-populations^23^. An AIM profile was included in the “Accepted” category if it was: (1) accepted in only one meta-population (z-score ≤ 1.64; *P* ≥ 0.05) or (2) accepted in more than one meta-population (z-score ≤ 1.64; *P* ≥ 0.05) and the likelihood of the AIM profile belonging to the population was statistically significantly higher than those of all other likelihoods (*P* < 0.05). An AIM profile was included in the “Rejected” category if it was not accepted in any meta-population (z-score > 1.64). An AIM profile was included in the “Ambiguous” category if it was (1) accepted in more than one meta-population (z-scores ≤ 1.64; *P* ≥ 0.05) and (2) the population likelihoods were not statistically significantly different from each other (z-scores ≤ 1.64; *P* ≥ 0.05). The likelihood ratios (LR) (Figs. 3, 4) were only calculated for individuals that were assigned “Accepted” or “Ambiguous”.

R v. 3.5.0 and the *‘ggplot2’* v. 3.2.1 R package (https://ggplot2.tidyverse.org/index.html) were used to visualise the LRs.

## Supplementary Information

Supplementary Information.

## References

[1] Kayser M, de Knijff P (2011). Improving human forensics through advances in genetics, genomics and molecular biology. Nat. Rev. Genet..

[2] Santos C (2016). Pacifiplex: An ancestry-informative SNP panel centred on Australia and the Pacific region. Forensic Sci. Int. Genet..

[3] Phillips C (2015). Forensic genetic analysis of bio-geographical ancestry. Forensic Sci. Int. Genet..

[4] Kidd KK (2014). Progress toward an efficient panel of SNPs for ancestry inference. Forensic Sci. Int. Genet..

[5] Nassir R (2009). An ancestry informative marker set for determining continental origin: validation and extension using human genome diversity panels. BMC Genet..

[6] Pereira V, Mogensen HS, Børsting C, Morling N (2017). Evaluation of the Precision ID Ancestry Panel for crime case work: A SNP typing assay developed for typing of 165 ancestral informative markers. Forensic Sci. Int. Genet..

[7] Themudo GE, Mogensen HS, Børsting C, Morling N (2016). Frequencies of HID-ion ampliseq ancestry panel markers among greenlanders. Forensic Sci. Int. Genet..

[8] Kosoy R (2009). Ancestry informative marker sets for determining continental origin and admixture proportions in common populations in America. Hum. Mutat..

[9] Jäger AC (2017). Developmental validation of the MiSeq FGx forensic genomics system for targeted next generation sequencing in forensic DNA casework and database laboratories. Forensic Sci. Int. Genet..

[10] Luis JR (2004). The Levant versus the horn of Africa: Evidence for bidirectional corridors of human migrations. Am. J. Hum. Genet..

[11] Bourke S (2018). The Middle East: The Cradle of Civilization.

[12] Haber M (2011). Influences of history, geography, and religion on genetic structure: the Maronites in Lebanon. Eur. J. Hum. Genet..

[13] Zalloua PA (2008). Y-chromosomal diversity in Lebanon is structured by recent historical events. Am. J. Hum. Genet..

[14] Zalloua PA (2008). Identifying genetic traces of historical expansions: Phoenician footprints in the Mediterranean. Am. J. Hum. Genet..

[15] Phillips C (2013). Eurasiaplex: A forensic SNP assay for differentiating European and South Asian ancestries. Forensic Sci. Int. Genet..

[16] Meier D (2018). Introduction to the special issue: Bordering the middle east. Geopolitics.

[17] Haber M (2013). Genome-wide diversity in the levant reveals recent structuring by culture. PLoS Genet..

[18] Thareja G (2015). Sequence and analysis of a whole genome from Kuwaiti population subgroup of Persian ancestry. BMC Genom..

[19] Bulbul O (2016). Inference of biogeographical ancestry across central regions of Eurasia. Int. J. Legal Med..

[20] Pereira V (2019). Development and validation of the EUROFORGEN NAME (North African and Middle Eastern) ancestry panel. Forensic Sci. Int. Genet..

[21] Truelsen DM, Pereira V, Phillips C, Morling N, Børsting C (2019). The EUROFORGEN NAME Ampliseq^TM^ custom panel: A second tier panel developed for differentiation of individuals from the Middle East/North Africa. Forensic Sci. Int. Genet. Suppl. Ser..

[22] Tvedebrink T, Eriksen PS, Mogensen HS, Morling N (2018). Weight of the evidence of genetic investigations of ancestry informative markers. Theor. Popul. Biol..

[23] Mogensen HS, Tvedebrink T, Børsting C, Pereira V, Morling N (2020). Ancestry prediction efficiency of the software GenoGeographer using a z-score method and the ancestry informative markers in the Precision ID Ancestry Panel. Forensic Sci. Int. Genet..

[24] Al-Asfi M (2018). Assessment of the precision ID ancestry panel. Int. J. Legal Med..

[25] Nakanishi H (2018). Analysis of mainland Japanese and Okinawan Japanese populations using the precision ID Ancestry Panel. Forensic Sci. Int. Genet..

[26] Hollard C (2017). Case report: on the use of the HID-Ion AmpliSeq^TM^ Ancestry Panel in a real forensic case. Int. J. Legal Med..

[27] García O (2017). Frequencies of the precision ID ancestry panel markers in Basques using the Ion Torrent PGM TM platform. Forensic Sci. Int. Genet..

[28] Lee JH (2018). Genetic resolution of applied biosystems^TM^ precision ID Ancestry panel for seven Asian populations. Leg. Med..

[29] Hussing C, Børsting C, Mogensen HS, Morling N (2015). Testing of the Illumina® ForenSeq^TM^ kit. Forensic Sci. Int. Genet. Suppl. Ser..

[30] Sharma V (2019). Evaluation of ForenSeq^TM^ Signature Prep Kit B on predicting eye and hair coloration as well as biogeographical ancestry by using Universal Analysis Software (UAS) and available web-tools. Electrophoresis.

[31] Truelsen DM (2017). Typing of two Middle Eastern populations with the Precision ID Ancestry Panel. Forensic Sci. Int. Genet. Suppl. Ser..

[32] Bulbul O, Cherni L, Khodjet-el-khil H, Rajeevan H, Kidd KK (2016). Evaluating a subset of ancestry informative SNPs for discriminating among Southwest Asian and circum-Mediterranean populations. Forensic Sci. Int. Genet..

[33] Bulbul O (2018). Improving ancestry distinctions among Southwest Asian populations. Forensic Sci. Int. Genet..

[34] Meyer OS, Andersen JD, Børsting C (2019). Presentation of the Human Pigmentation (HuPi) AmpliSeq^TM^ custom panel. Forensic Sci. Int. Genet. Suppl. Ser..

[35] Kersbergen P (2009). Developing a set of ancestry-sensitive DNA markers reflecting continental origins of humans. BMC Genet..

[36] Morling N (2002). Paternity testing commission of the international society of forensic genetics. Int. J. Legal Med..

[37] Gill P (2006). DNA commission of the International Society of Forensic Genetics: Recommendations on the interpretation of mixtures. Forensic Sci. Int..

[38] van der Heijden S, de Oliveira SJ, Kampmann M-L, Børsting C, Morling N (2017). Comparison of manual and automated AmpliSeq^TM^ workflows in the typing of a Somali population with the Precision ID Identity Panel. Forensic Sci. Int. Genet..

[39] Farzad MS, Pedersen BM, Mogensen HS, Børsting C (2020). Development of an automated AmpliSeq^TM^ library building workflow for biological stain samples on the Biomek ® 3000. Biotechniques.

[40] Excoffier L, Lischer HEL (2010). Arlequin suite ver 3.5: a new series of programs to perform population genetics analyses under Linux and Windows. Mol. Ecol. Resour..

[41] Bonferroni, C. E. Teoria statistica delle classi e calcolo delle probabilità. *Pubbl. del R Ist. Super. di Sci. Econ. e Commer. di Firenze* (1936).

[42] Gabriel SB (2002). The structure of haplotype blocks in the human genome. Science (80-).

[43] Jombart T (2008). adegenet: a R package for the multivariate analysis of genetic markers. Bioinformatics.

[44] Dray S, Dufour A-B (2007). The ade4 package: Implementing the duality diagram for ecologists. J. Stat. Softw..

[45] Pritchard JK, Stephens M, Donnelly P (2000). Inference of population structure using multilocus genotype data. Genetics.

[46] Falush D, Stephens M, Pritchard JK (2003). Inference of population structure using multilocus genotype data: Linked loci and correlated allele frequencies. Genetics.

[47] Earl DA, VonHoldt BM (2012). STRUCTURE HARVESTER: a website and program for visualizing STRUCTURE output and implementing the Evanno method. Conserv. Genet. Resour..

[48] Jakobsson M, Rosenberg NA (2007). CLUMPP: A cluster matching and permutation program for dealing with label switching and multimodality in analysis of population structure. Bioinformatics.

[49] Rosenberg NA (2003). distruct: A program for the graphical display of population structure. Mol. Ecol. Notes.

[50] Chakraborty R, Srinivasan MR, Daiger SP (1993). Evaluation of standard error and confidence interval of estimated multilocus genotype probabilities, and their implications in DNA forensics. Am. J. Hum. Genet..

