# Supplementary material for: Assessment of the effectiveness of the EUROFORGEN NAME and Precision ID Ancestry panel markers for ancestry investigations
Source: Sci Rep. 2021 Sep 20;11:18595. doi: 10.1038/s41598-021-97654-0 (PMC8452675; doi:10.1038/s41598-021-97654-0)
Supplement: Supplementary file 1 — Supplementary Information. [file 41598_2021_97654_MOESM1_ESM.docx]

**Supplementary materials**

D. M. Truelsen^a^, T. Tvedebrink^a,b^, H. S. Mogensen^a^, M. S. Farzad^a^, M. A. Shan^a^, N. Morling^a,b^, V. Pereira^a^, C. Børsting^a^

*Assessment of the effectiveness of the EUROFORGEN NAME and Precision ID Ancestry panel markers for ancestry investigations*

^a^ Section of Forensic Genetics, Department of Forensic Medicine, Faculty of Health and Medical Sciences, University of Copenhagen, 2100 Copenhagen, Denmark

^b^ Department of Mathematical Sciences, Aalborg University, 9220 Aalborg, Denmark

Table S1: SNPs included in the EUROFORGEN NAME panel.

| **Chromosome** | **Position (hg19)** | **rs-number** |
| --- | --- | --- |
| chr1 | 170700758 | rs1476040 |
| chr1 | 172879023 | rs2227203 |
| chr1 | 170642899 | rs593479 |
| chr1 | 236568770 | rs608825 |
| chr2 | 135332355 | rs10928499 |
| chr2 | 135637338 | rs2166480 |
| chr2 | 135730884 | rs2874714 |
| chr2 | 135691659 | rs4344970 |
| chr2 | 135295180 | rs621341 |
| chr2 | 135305510 | rs6430524 |
| chr2 | 38652572 | rs6741107 |
| chr2 | 136928320 | rs7568884 |
| chr2 | 135837906 | rs7570971 |
| chr2 | 136825272 | rs932206 |
| chr3 | 65227760 | rs1471504 |
| chr3 | 65226990 | rs17349171 |
| chr3 | 465476 | rs6805861 |
| chr3 | 197083723 | rs718501 |
| chr3 | 181592674 | rs734482 |
| chr3 | 76473163 | rs9817359 |
| chr3 | 65762252 | rs984038 |
| chr4 | 38745482 | rs10008032 |
| chr4 | 123997900 | rs12508176 |
| chr4 | 130031498 | rs1699387 |
| chr4 | 130022161 | rs1757928 |
| chr4 | 38812876 | rs17616434 |
| chr4 | 130030652 | rs337277 |
| chr4 | 156688928 | rs3796564 |
| chr4 | 38815502 | rs4833103 |
| chr4 | 119980752 | rs4834738 |
| chr4 | 129980630 | rs4975193 |
| chr4 | 129757215 | rs4975270 |
| chr5 | 127682184 | rs11746746 |
| chr5 | 56718875 | rs2172771 |
| chr5 | 136334314 | rs4308478 |
| chr6 | 73733188 | rs11963228 |

Table S1 continued

| **Chromosome** | **Position (hg19)** | **rs-number** |
| --- | --- | --- |
| chr6 | 91772347 | rs11963862 |
| chr6 | 36490733 | rs12203115 |
| chr6 | 161154955 | rs1317026 |
| chr6 | 145004411 | rs1406045 |
| chr6 | 124210612 | rs17086288 |
| chr6 | 161144729 | rs1853025 |
| chr6 | 24972729 | rs387070 |
| chr6 | 145097563 | rs6901197 |
| chr6 | 145030284 | rs9321987 |
| chr6 | 73757091 | rs9342997 |
| chr7 | 112353440 | rs10215501 |
| chr7 | 22774231 | rs10242595 |
| chr7 | 112411379 | rs1227171 |
| chr7 | 112360669 | rs1922897 |
| chr7 | 112318992 | rs2028028 |
| chr7 | 112334945 | rs2966487 |
| chr7 | 112516977 | rs6973324 |
| chr7 | 112464841 | rs821785 |
| chr7 | 77582265 | rs848461 |
| chr7 | 112392941 | rs9649356 |
| chr8 | 15298515 | rs1495085 |
| chr8 | 59872177 | rs1947178 |
| chr8 | 101680355 | rs2935548 |
| chr8 | 59925618 | rs310362 |
| chr8 | 54701811 | rs4737753 |
| chr8 | 128643231 | rs6989963 |
| chr8 | 110413762 | rs964307 |
| chr9 | 78158285 | rs11144480 |
| chr9 | 110452511 | rs1403856 |
| chr9 | 110454145 | rs2049366 |
| chr9 | 138603740 | rs487750 |
| chr9 | 753093 | rs7025610 |
| chr9 | 137098042 | rs7872110 |
| chr9 | 137112655 | rs7873963 |
| chr10 | 15674005 | rs12414926 |
| chr10 | 56847522 | rs1733743 |
| chr10 | 14815820 | rs1974068 |
| chr10 | 4069527 | rs2031581 |
| chr10 | 4074096 | rs2765650 |
| chr10 | 102075479 | rs603424 |
| chr10 | 121965926 | rs7078514 |

Table S1 continued

| **Chromosome** | **Position (hg19)** | **rs-number** |
| --- | --- | --- |
| chr11 | 104955489 | rs1785864 |
| chr11 | 83507759 | rs4396309 |
| chr11 | 104991640 | rs4587693 |
| chr11 | 17572284 | rs7102164 |
| chr11 | 109241241 | rs7947221 |
| chr12 | 83205152 | rs10506882 |
| chr12 | 26390761 | rs10743577 |
| chr12 | 26428793 | rs7975017 |
| chr12 | 83201596 | rs10862511 |
| chr14 | 74212431 | rs10131666 |
| chr14 | 68236522 | rs2295111 |
| chr14 | 65680885 | rs7148809 |
| chr15 | 28386626 | rs11636232 |
| chr15 | 28365618 | rs12913832 |
| chr15 | 28530182 | rs1667394 |
| chr15 | 100285705 | rs8027443 |
| chr16 | 4083105 | rs6500567 |
| chr16 | 8454655 | rs896401 |
| chr17 | 59584565 | rs10853034 |
| chr17 | 53757787 | rs1549519 |
| chr17 | 53248927 | rs1564821 |
| chr17 | 58361461 | rs8064588 |
| chr17 | 36185665 | rs9899480 |
| chr18 | 66810095 | rs17080463 |
| chr18 | 20875793 | rs2337024 |
| chr20 | 57488964 | rs234623 |
| chr20 | 54085557 | rs3852929 |
| chr20 | 2035775 | rs6081765 |
| chr21 | 37133457 | rs2835133 |
| chr21 | 37301530 | rs2835162 |

Table S2: Reference populations whose AIM profiles were compared to those obtained with the EUROFORGEN NAME panel. The AIM data were obtained from the1000 Genomes Project, the HGDP-CEPH panel, and this study.

| **Population** | **Abbreviation** | **Population size** |
| --- | --- | --- |
| Esan in Nigeria | ESN | 99 |
| Gambian in Western Divisions in the Gambia | GWD | 113 |
| Luhya in Webuye, Kenya | LWK | 99 |
| Mende in Sierra Leone | MSL | 85 |
| Yoruba in Ibadan, Nigeria | YRI | 108 |
| Chinese Dai in Xishuangbanna, China | CDX | 93 |
| Han Chinese in Bejing, China | CHB | 103 |
| Southern Han Chinese | HCS | 105 |
| Japanese in Tokyo, Japan | JPT | 104 |
| Kinh in Ho Chi Minh City, Vietnam | KHV | 99 |
| Bengali from Bangladesh | BEB | 86 |
| Gujarati Indian from Houston, Texas | GIH | 103 |
| Indian Telugu from the UK | ITU | 102 |
| Punjabi from Lahore, Pakistan | PJL | 96 |
| Sri Lankan Tamil from the UK | STU | 102 |
| British in England and Scotland | GBR | 91 |
| Finnish in Finland | FN1 | 99 |
| Toscani in Italy | TSI | 107 |
| Iberian Population in Spain | IBS | 107 |
| N&W European Utah Residents (CEPH) | CEU | 99 |
| France – Basque | FRB | 24 |
| France – French | FRN | 28 |
| Israel (Carmel) – Druze | DRU | 42 |
| Israel (Central) – Palestinian | PLA | 46 |
| Israel (Negev) – Bedouin | BDN | 46 |
| Pakistan – Balochi | BCH | 24 |
| Pakistan – Kalash | KLS | 23 |
| Algeria (Mzab) – Mozabite | MOZ | 29 |
| Libya | LIB | 47 |
| Morocco | MOR1 | 25 |
| Morocco (This study) | MOR2 | 75 |
| Afghanistan | AFG1 | 47 |
| Afghanistan (This study) | AFG2 | 67 |
| Kuwait | KUW | 43 |
| Turkey | TUR1 | 30 |
| Turkey (This study) | TUR2 | 79 |
| Iraq (This study) | IRQ | 72 |
| Iran (This study) | IRN | 77 |
| Syria (This study) | SYR | 79 |
| Portugal (This study) | POR | 79 |
| Denmark (This study) | DEN | 79 |
| Albania (This study) | ALB | 80 |

Table S2 continued

| **Population** | **Abbreviation** | **Population size** |
| --- | --- | --- |
| Greece (This study) | GRE | 79 |
| Slovenia (This study) | SLO | 81 |
| Pakistan (This study) | PUN | 77 |
| Indian | IND | 23 |
| Azerbaijan | AZE | 15 |
| Eritrea (This study) | ERI | 79 |
| Somalia (This study) | SOM | 70 |

Table S3: Reference populations whose AIM profiles were compared to those obtained with both the AmpliSeq Ancestry ID Panel and the EUROFORGEN NAME panel. The AIM data were obtained from the1000 Genomes Project, the HGDP-CEPH panel, and this study.

| **Population** | **Abbreviation** | **Population size** |
| --- | --- | --- |
| Esan in Nigeria | ESN | 99 |
| Gambian in Western Divisions in the Gambia | GWD | 113 |
| Luhya in Webuye, Kenya | LWK | 99 |
| Mende in Sierra Leone | MSL | 85 |
| Yoruba in Ibadan, Nigeria | YRI | 108 |
| Chinese Dai in Xishuangbanna, China | CDX | 93 |
| Han Chinese in Bejing, China | CHB | 103 |
| Southern Han Chinese | HCS | 105 |
| Japanese in Tokyo, Japan | JPT | 104 |
| Kinh in Ho Chi Minh City, Vietnam | KHV | 99 |
| Bengali from Bangladesh | BEB | 86 |
| Gujarati Indian from Houston, Texas | GIH | 103 |
| Indian Telugu from the UK | ITU | 102 |
| Punjabi from Lahore, Pakistan | PJL | 96 |
| Sri Lankan Tamil from the UK | STU | 102 |
| British in England and Scotland | GBR | 91 |
| Finnish in Finland | FN1 | 99 |
| Toscani in Italy | TSI | 107 |
| Iberian Population in Spain | IBS | 107 |
| N&W European Utah Residents (CEPH) | CEU | 99 |
| Morocco (This study) | MOR2 | 75 |
| Afghanistan (This study) | AFG2 | 67 |
| Turkey (This study) | TUR2 | 79 |
| Iraq (This study) | IRQ | 72 |
| Iran (This study) | IRN | 77 |
| Syria (This study) | SYR | 79 |
| Portugal (This study) | POR | 79 |
| Denmark (This study) | DEN | 79 |
| Albania (This study) | ALB | 80 |
| Greece (This study) | GRE | 79 |
| Slovenia (This study) | SLO | 81 |
| Pakistan (This study) | PUN | 77 |
| Eritrea (This study) | ERI | 79 |
| Somalia (This study) | SOM | 70 |

Table S4: Loci in LD and situated on the same chromosome.

| **Locus 1** | | | | | **Locus 2** | | | | |  |  |
| --- | --- | --- | --- | --- | --- | --- | --- | --- | --- | --- | --- |
| **Chromosome** | **Position** | **rs-number** | **Panel** | **cM** | **Chromosome** | **Position** | **rs-number** | **Panel** | **cM** | **Distance bp** | **Distance cM** |
| chr10 | 4069527 | rs2031581 | NAME | 11.99 | chr10 | 4074096 | rs2765650 | NAME | 12.01 | 4569 | 0.021 |
| chr11 | 104955489 | rs1785864 | NAME | 110.98 | chr11 | 104991640 | rs4587693 | NAME | 110.99 | 36151 | 0.012 |
| chr12 | 83205152 | rs10506882 | NAME | 98.77 | chr12 | 83201596 | rs10862511 | NAME | 98.77 | 3556 | 0.003 |
| chr15 | 28386626 | rs11636232 | NAME | 16.09 | chr15 | 28365618 | rs12913832 | NAME | 16.02 | 21008 | 0.071 |
| chr15 | 28365618 | rs12913832 | NAME | 16.02 | chr15 | 28530182 | rs1667394 | NAME | 16.55 | 164564 | 0.532 |
| chr17 | 53757787 | rs1549519 | NAME | 82.50 | chr17 | 53788280 | rs2033111 | Precision | 82.56 | 30493 | 0.056 |
| chr18 | 67578931 | rs3916235 | Precision | 103.90 | chr18 | 67867663 | rs4891825 | Precision | 104.30 | 288732 | 0.396 |
| chr2 | 135332355 | rs10928499 | NAME | 147.29 | chr2 | 135305510 | rs6430524 | NAME | 147.26 | 26845 | 0.032 |
| chr2 | 135637338 | rs2166480 | NAME | 147.63 | chr2 | 135730884 | rs2874714 | NAME | 147.70 | 93546 | 0.074 |
| chr2 | 135637338 | rs2166480 | NAME | 147.63 | chr2 | 135691659 | rs4344970 | NAME | 147.67 | 54321 | 0.042 |
| chr2 | 135637338 | rs2166480 | NAME | 147.63 | chr2 | 135295180 | rs621341 | NAME | 147.24 | 342158 | 0.387 |
| chr2 | 135637338 | rs2166480 | NAME | 147.63 | chr2 | 136707982 | rs6754311 | Precision | 148.23 | 1070644 | 0.597 |
| chr2 | 135637338 | rs2166480 | NAME | 147.63 | chr2 | 136928320 | rs7568884 | NAME | 148.34 | 1290982 | 0.712 |
| chr2 | 135637338 | rs2166480 | NAME | 147.63 | chr2 | 135837906 | rs7570971 | NAME | 147.79 | 200568 | 0.162 |
| chr2 | 135637338 | rs2166480 | NAME | 147.63 | chr2 | 136825272 | rs932206 | NAME | 148.29 | 1187934 | 0.655 |
| chr2 | 135730884 | rs2874714 | NAME | 147.70 | chr2 | 135691659 | rs4344970 | NAME | 147.67 | 39225 | 0.032 |
| chr2 | 135295180 | rs621341 | NAME | 147.24 | chr2 | 136707982 | rs6754311 | Precision | 148.23 | 1412802 | 0.984 |
| chr2 | 135295180 | rs621341 | NAME | 147.24 | chr2 | 135837906 | rs7570971 | NAME | 147.79 | 542726 | 0.548 |
| chr2 | 136707982 | rs6754311 | Precision | 148.23 | chr2 | 135837906 | rs7570971 | NAME | 147.79 | 870076 | 0.435 |
| chr2 | 136707982 | rs6754311 | Precision | 148.23 | chr2 | 136825272 | rs932206 | NAME | 148.29 | 117290 | 0.059 |
| chr2 | 136928320 | rs7568884 | NAME | 148.34 | chr2 | 136825272 | rs932206 | NAME | 148.29 | 103048 | 0.057 |
| chr2 | 135837906 | rs7570971 | NAME | 147.79 | chr2 | 136825272 | rs932206 | NAME | 148.29 | 987366 | 0.494 |
| chr21 | 37133457 | rs2835133 | NAME | 42.46 | chr21 | 37301530 | rs2835162 | NAME | 42.89 | 168073 | 0.434 |
| chr3 | 65227760 | rs1471504 | NAME | 88.34 | chr3 | 65226990 | rs17349171 | NAME | 88.33 | 770 | 0.001 |
| chr4 | 38745482 | rs10008032 | NAME | 57.67 | chr4 | 38812876 | rs17616434 | NAME | 57.75 | 67394 | 0.074 |
| chr4 | 130031498 | rs1699387 | NAME | 132.73 | chr4 | 130022161 | rs1757928 | NAME | 132.72 | 9337 | 0.008 |

Table S4 continued

| **Locus 1** | | | | | **Locus 2** | | | | |  |  |
| --- | --- | --- | --- | --- | --- | --- | --- | --- | --- | --- | --- |
| **Chromosome** | **Position** | **rs-number** | **Panel** | **cM** | **Chromosome** | **Position** | **rs-number** | **Panel** | **cM** | **Distance bp** | **Distance cM** |
| chr4 | 130031498 | rs1699387 | NAME | 132.73 | chr4 | 130030652 | rs337277 | NAME | 132.72 | 846 | 0.001 |
| chr4 | 130031498 | rs1699387 | NAME | 132.73 | chr4 | 129980630 | rs4975193 | NAME | 132.68 | 50868 | 0.046 |
| chr4 | 130031498 | rs1699387 | NAME | 132.73 | chr4 | 129757215 | rs4975270 | NAME | 132.49 | 274283 | 0.237 |
| chr4 | 130022161 | rs1757928 | NAME | 132.72 | chr4 | 130030652 | rs337277 | NAME | 132.72 | 8491 | 0.008 |
| chr4 | 130022161 | rs1757928 | NAME | 132.72 | chr4 | 129980630 | rs4975193 | NAME | 132.68 | 41531 | 0.037 |
| chr4 | 130022161 | rs1757928 | NAME | 132.72 | chr4 | 129757215 | rs4975270 | NAME | 132.49 | 264946 | 0.228 |
| chr4 | 38812876 | rs17616434 | NAME | 57.75 | chr4 | 38815502 | rs4833103 | NAME | 57.75 | 2626 | 0.003 |
| chr4 | 130030652 | rs337277 | NAME | 132.72 | chr4 | 129980630 | rs4975193 | NAME | 132.68 | 50022 | 0.045 |
| chr4 | 130030652 | rs337277 | NAME | 132.72 | chr4 | 129757215 | rs4975270 | NAME | 132.49 | 273437 | 0.236 |
| chr4 | 129980630 | rs4975193 | NAME | 132.68 | chr4 | 129757215 | rs4975270 | NAME | 132.49 | 223415 | 0.191 |
| chr6 | 73733188 | rs11963228 | NAME | 90.90 | chr6 | 73757091 | rs9342997 | NAME | 90.92 | 23903 | 0.020 |
| chr6 | 145004411 | rs1406045 | NAME | 152.05 | chr6 | 145055331 | rs4463276 | Precision | 152.09 | 50920 | 0.034 |
| chr6 | 145004411 | rs1406045 | NAME | 152.05 | chr6 | 145097563 | rs6901197 | NAME | 152.12 | 93152 | 0.066 |
| chr6 | 145004411 | rs1406045 | NAME | 152.05 | chr6 | 145030284 | rs9321987 | NAME | 152.07 | 25873 | 0.017 |
| chr6 | 145055331 | rs4463276 | Precision | 152.09 | chr6 | 145097563 | rs6901197 | NAME | 152.12 | 42232 | 0.032 |
| chr6 | 145055331 | rs4463276 | Precision | 152.09 | chr6 | 145030284 | rs9321987 | NAME | 152.07 | 25047 | 0.017 |
| chr6 | 145097563 | rs6901197 | NAME | 152.12 | chr6 | 145030284 | rs9321987 | NAME | 152.07 | 67279 | 0.050 |
| chr7 | 112353440 | rs10215501 | NAME | 121.75 | chr7 | 112411379 | rs1227171 | NAME | 121.78 | 57939 | 0.030 |
| chr7 | 112353440 | rs10215501 | NAME | 121.75 | chr7 | 112318992 | rs2028028 | NAME | 121.73 | 34448 | 0.017 |
| chr7 | 112353440 | rs10215501 | NAME | 121.75 | chr7 | 112516977 | rs6973324 | NAME | 121.84 | 163537 | 0.087 |
| chr7 | 112353440 | rs10215501 | NAME | 121.75 | chr7 | 112464841 | rs821785 | NAME | 121.81 | 111401 | 0.059 |
| chr7 | 112353440 | rs10215501 | NAME | 121.75 | chr7 | 112392941 | rs9649356 | NAME | 121.77 | 39501 | 0.020 |
| chr7 | 112411379 | rs1227171 | NAME | 121.78 | chr7 | 112318992 | rs2028028 | NAME | 121.73 | 92387 | 0.047 |
| chr7 | 112411379 | rs1227171 | NAME | 121.78 | chr7 | 112516977 | rs6973324 | NAME | 121.84 | 105598 | 0.057 |
| chr7 | 112411379 | rs1227171 | NAME | 121.78 | chr7 | 112464841 | rs821785 | NAME | 121.81 | 53462 | 0.029 |
| chr7 | 112411379 | rs1227171 | NAME | 121.78 | chr7 | 112392941 | rs9649356 | NAME | 121.77 | 18438 | 0.010 |

Table S4 continued

| **Locus 1** | | | | | **Locus 2** | | | | |  |  |
| --- | --- | --- | --- | --- | --- | --- | --- | --- | --- | --- | --- |
| **Chromosome** | **Position** | **rs-number** | **Panel** | **cM** | **Chromosome** | **Position** | **rs-number** | **Panel** | **cM** | **Distance bp** | **Distance cM** |
| chr7 | 112318992 | rs2028028 | NAME | 121.73 | chr7 | 112516977 | rs6973324 | NAME | 121.84 | 197985 | 0.104 |
| chr7 | 112318992 | rs2028028 | NAME | 121.73 | chr7 | 112464841 | rs821785 | NAME | 121.81 | 145849 | 0.076 |
| chr7 | 112318992 | rs2028028 | NAME | 121.73 | chr7 | 112392941 | rs9649356 | NAME | 121.77 | 73949 | 0.037 |
| chr7 | 112516977 | rs6973324 | NAME | 121.84 | chr7 | 112464841 | rs821785 | NAME | 121.81 | 52136 | 0.028 |
| chr7 | 112516977 | rs6973324 | NAME | 121.84 | chr7 | 112392941 | rs9649356 | NAME | 121.77 | 124036 | 0.066 |
| chr7 | 112464841 | rs821785 | NAME | 121.81 | chr7 | 112392941 | rs9649356 | NAME | 121.77 | 71900 | 0.038 |
| chr8 | 59872177 | rs1947178 | NAME | 74.23 | chr8 | 59925618 | rs310362 | NAME | 74.28 | 53441 | 0.056 |
| chr9 | 110452511 | rs1403856 | NAME | 113.30 | chr9 | 110454145 | rs2049366 | NAME | 113.30 | 1634 | 0.004 |
| chr9 | 137098042 | rs7872110 | NAME | 153.53 | chr9 | 137112655 | rs7873963 | NAME | 153.62 | 14613 | 0.094 |

Table S5: Loci in LD and situated on different chromosomes.

|  | **Locus 1** | | | | | **Locus 2** | | | |
| --- | --- | --- | --- | --- | --- | --- | --- | --- | --- |
| **Population** | **Chromo-some** | **Position** | **rs-number** | **cM** | **Chromo-some** | | **Position** | **rs-number** | **cM** |
| Afghanistan | chr17 | 53788280 | rs2033111 | 82.56 | chr3 | | 65762252 | rs984038 | 89.26 |
| Afghanistan | chr2 | 135305510 | rs6430524 | 147.26 | chr16 | | 4083105 | rs6500567 | 9.87 |
| Afghanistan | chr2 | 29538411 | rs4666200 | 50.29 | chr18 | | 67867663 | rs4891825 | 104.30 |
| Afghanistan | chr22 | 18076546 | rs1296819 | 6.26 | chr2 | | 135305510 | rs6430524 | 147.26 |
| Albania | chr1 | 168159890 | rs1040404 | 178.55 | chr10 | | 56847522 | rs1733743 | 72.82 |
| Albania | chr17 | 73782191 | rs2125345 | 114.86 | chr18 | | 20875793 | rs2337024 | 48.80 |
| Albania | chr4 | 100239319 | rs1229984 | 109.98 | chr1 | | 212786883 | rs4951629 | 222.04 |
| Albania | chr5 | 165739982 | rs1500127 | 175.79 | chr15 | | 74734500 | rs2899826 | 77.96 |
| Denmark | chr3 | 179964727 | rs2030763 | 186.80 | chr7 | | 112516977 | rs6973324 | 121.84 |
| Denmark | chr7 | 112411379 | rs1227171 | 121.78 | chr4 | | 38812876 | rs17616434 | 57.75 |
| Eritrea | chr1 | 172879023 | rs2227203 | 183.51 | chr18 | | 9420504 | rs4798812 | 34.16 |
| Eritrea | chr1 | 18170886 | rs647325 | 39.40 | chr3 | | 135914476 | rs9845457 | 144.95 |
| Eritrea | chr14 | 105679055 | rs3784230 | 122.63 | chr1 | | 236568770 | rs608825 | 251.83 |
| Eritrea | chr2 | 135637338 | rs2166480 | 147.63 | chr6 | | 145097563 | rs6901197 | 152.12 |
| Eritrea | chr8 | 59925618 | rs310362 | 74.28 | chr2 | | 135691659 | rs4344970 | 147.67 |
| Greece | chr2 | 79864923 | rs13400937 | 104.48 | chr1 | | 172879023 | rs2227203 | 183.51 |
| Greece | chr4 | 38815502 | rs4833103 | 57.75 | chr6 | | 21911616 | rs7745461 | 45.84 |
| Iran | chr10 | 121965926 | rs7078514 | 142.56 | chr13 | | 27624356 | rs9319336 | 17.46 |
| Iran | chr6 | 73733188 | rs11963228 | 90.90 | chr8 | | 59925618 | rs310362 | 74.28 |
| Iran | chr8 | 140241181 | rs2001907 | 159.89 | chr9 | | 137098042 | rs7872110 | 153.53 |
| Morocco | chr18 | 67578931 | rs3916235 | 103.90 | chr3 | | 135914476 | rs9845457 | 144.95 |
| Morocco | chr2 | 135730884 | rs2874714 | 147.70 | chr8 | | 122908503 | rs7844723 | 126.48 |
| Morocco | chr5 | 170202984 | rs7722456 | 185.63 | chr6 | | 73757091 | rs9342997 | 90.92 |
| Pakistan | chr10 | 4074096 | rs2765650 | 12.01 | chr1 | | 242342504 | rs316873 | 267.72 |
| Pakistan | chr18 | 49781544 | rs7238445 | 77.96 | chr3 | | 147750355 | rs734873 | 156.36 |
| Portugal | chr10 | 14815820 | rs1974068 | 36.14 | chr18 | | 67867663 | rs4891825 | 104.30 |
| Portugal | chr16 | 740466 | rs4984913 | 2.45 | chr20 | | 2035775 | rs6081765 | 7.50 |
| Portugal | chr4 | 179399523 | rs2702414 | 183.11 | chr6 | | 145097563 | rs6901197 | 152.12 |
| Portugal | chr5 | 165739982 | rs1500127 | 175.79 | chr7 | | 112318992 | rs2028028 | 121.73 |
| Portugal | chr5 | 127682184 | rs11746746 | 137.44 | chr8 | | 13359500 | rs3943253 | 26.74 |
| Somalia | chr12 | 26390761 | rs10743577 | 51.04 | chr5 | | 56718875 | rs2172771 | 72.95 |
| Syria | chr9 | 138603740 | rs487750 | 162.24 | chr2 | | 14756349 | rs7421394 | 32.13 |

Table S6: Loci that were excluded from further analysis due to LD with other loci.

| **Chromosome** | **Position (hg19)** | **rs-number** | **Length** | **Reason for exclusion** |
| --- | --- | --- | --- | --- |
| chr4 | 38745482 | rs10008032 | 127 | Longest amplicon size |
| chr7 | 112353440 | rs10215501 | 124 | Longest amplicon size |
| chr12 | 83201596 | rs10862511 | 113 | Longest amplicon size and poorest LB |
| chr2 | 135332355 | rs10928499 | 128 | Longest amplicon size |
| chr15 | 28386626 | rs11636232 | 113 | Longest amplicon size |
| chr6 | 73733188 | rs11963228 | 122 | Highest noise |
| chr7 | 112411379 | rs1227171 | 119 | Longest amplicon size |
| chr6 | 145004411 | rs1406045 | 122 | Longest amplicon size |
| chr17 | 53757787 | rs1549519 | 124 | Longest amplicon size |
| chr15 | 28530182 | rs1667394 | 122 | Longest amplicon size |
| chr4 | 130031498 | rs1699387 | 129 | Longest amplicon size |
| chr3 | 65226990 | rs17349171 | 130 | Longest amplicon size |
| chr4 | 130022161 | rs1757928 | 115 | Performed poorly with AmpliSeq |
| chr8 | 59872177 | rs1947178 | 120 | Longest amplicon size |
| chr7 | 112318992 | rs2028028 | 120 | Longest amplicon size |
| chr10 | 4069527 | rs2031581 | 127 | Longest amplicon size |
| chr9 | 110454145 | rs2049366 | 118 | Longest amplicon size and poorest LB |
| chr2 | 135637338 | rs2166480 | 120 | Longest amplicon size |
| chr21 | 37133457 | rs2835133 | 127 | Longest amplicon size and poorest LB |
| chr2 | 135730884 | rs2874714 | 114 | Longest amplicon size |
| chr4 | 130030652 | rs337277 | 116 | Longest amplicon size |
| chr6 | 145055331 | rs4463276 | 82 | NAME markers preferred over Precision ID markers |
| chr11 | 104991640 | rs4587693 | 126 | Performed poorly with MassARRAY |
| chr4 | 38815502 | rs4833103 | 127 | Longest amplicon size |
| chr4 | 129980630 | rs4975193 | 118 | Longest amplicon size |
| chr2 | 136707982 | rs6754311 | 60 | NAME markers preferred over Precision ID markers |
| chr6 | 145097563 | rs6901197 | 126 | Longest amplicon size |
| chr7 | 112516977 | rs6973324 | 119 | Longest amplicon size |
| chr2 | 135837906 | rs7570971 | 120 | Longest amplicon size |
| chr9 | 137112655 | rs7873963 | 126 | Longest amplicon size and poorest LB |
| chr7 | 112464841 | rs821785 | 120 | Longest amplicon size |
| chr2 | 136825272 | rs932206 | 124 | Longest amplicon size |


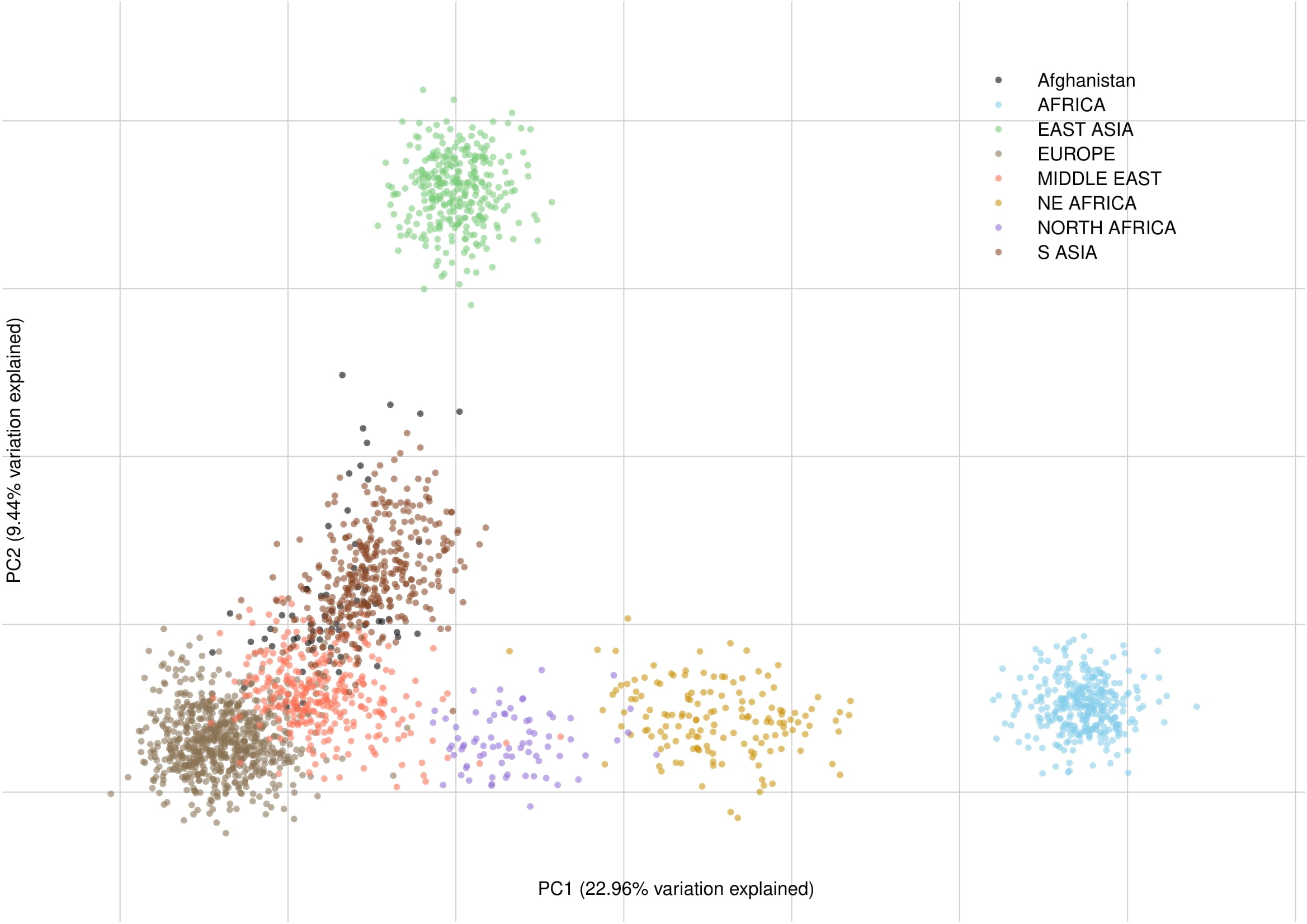


Figure S1: PCA plot of the results obtained with the combined dataset of 233 AIMs included in the EUROFORGEN NAME panel and the Precision ID Ancestry Panel for the Afghanistan individuals.


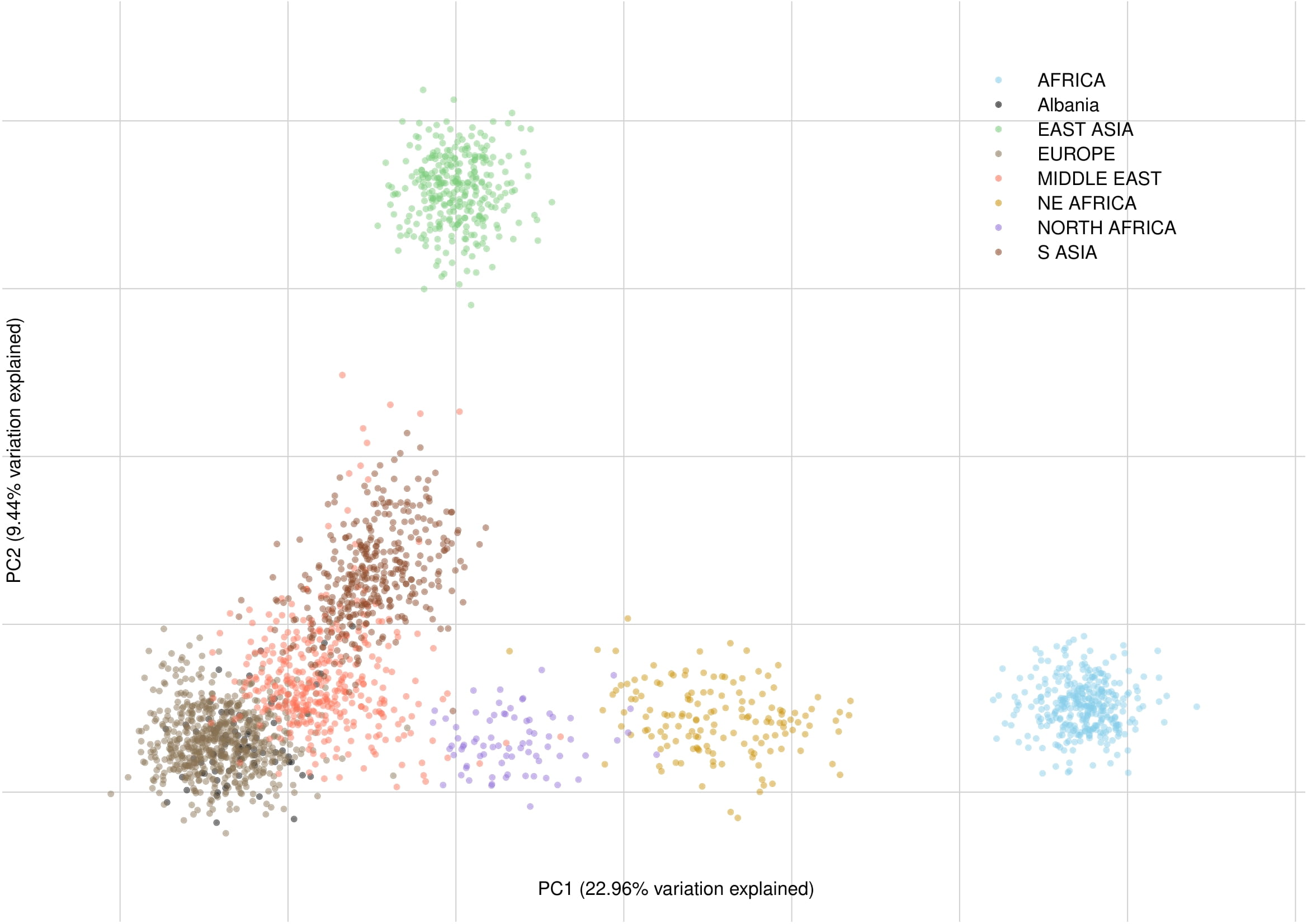


Figure S2: PCA plot of the results obtained with the combined dataset of 233 AIMs included in the EUROFORGEN NAME panel and the Precision ID Ancestry Panel for the Albanian individuals.


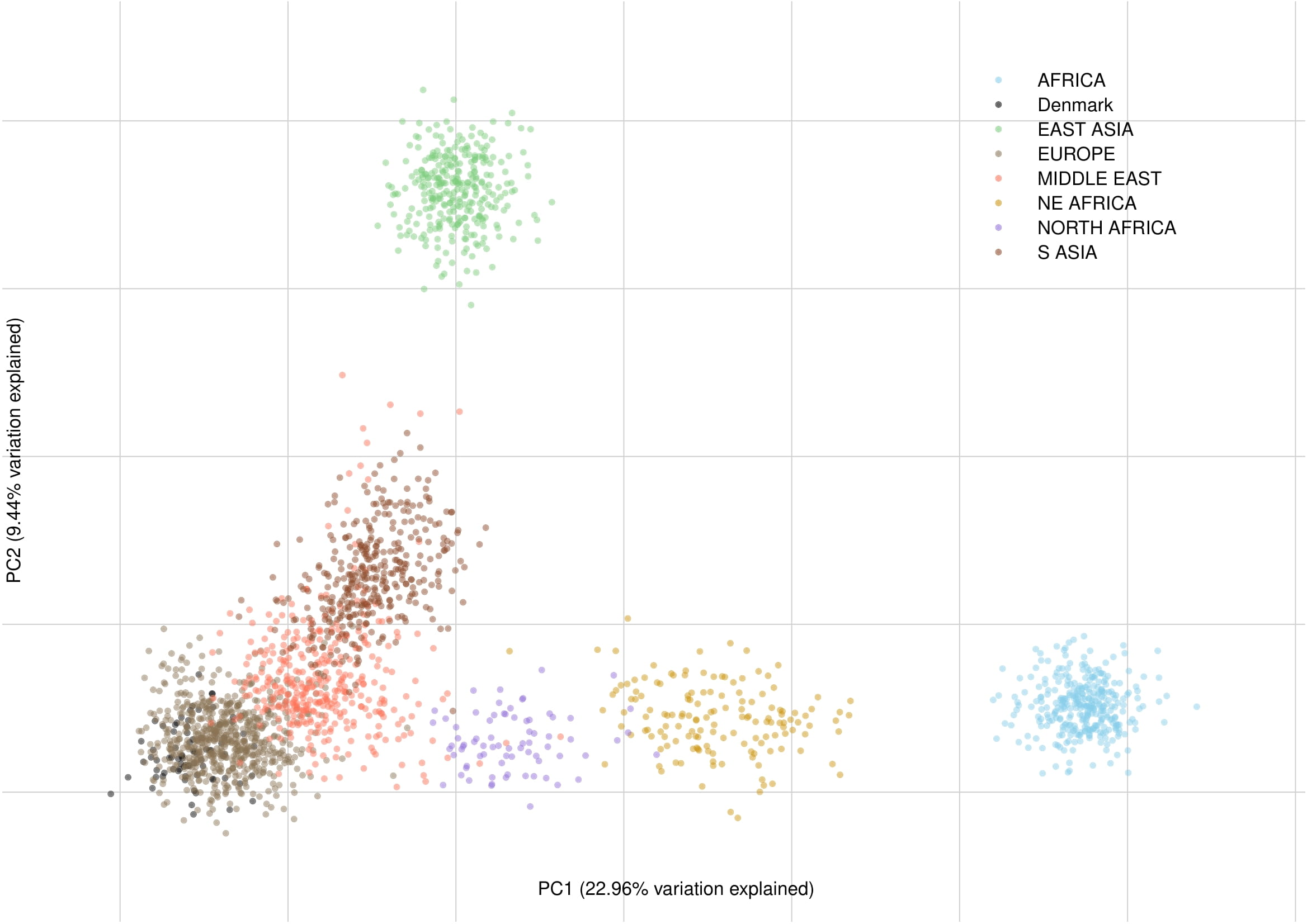


Figure S3: PCA plot of the results obtained with the combined dataset of 233 AIMs included in the EUROFORGEN NAME panel and the Precision ID Ancestry Panel for the Danish individuals.


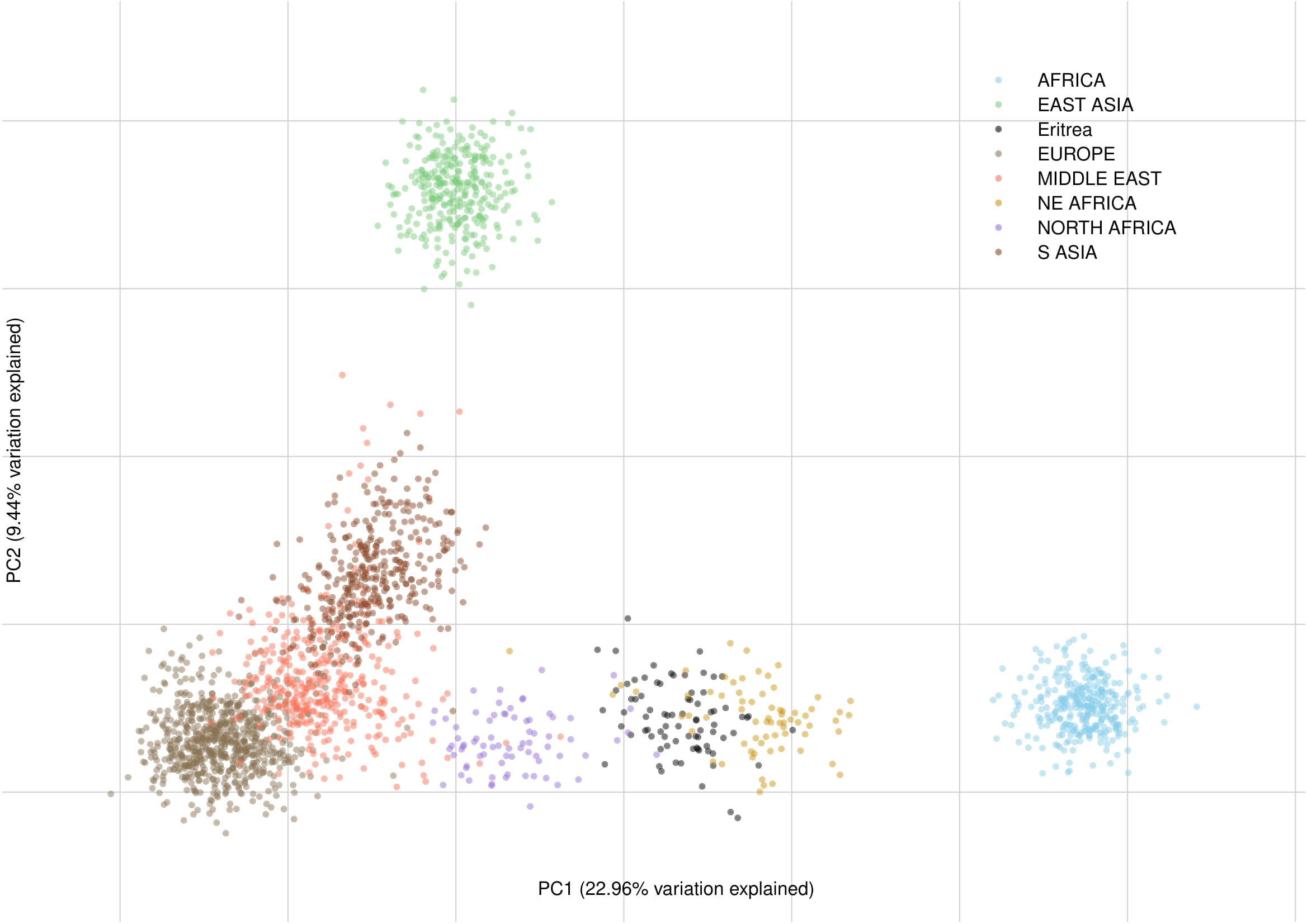


Figure S4: PCA plot of the results obtained with the combined dataset of 233 AIMs included in the EUROFORGEN NAME panel and the Precision ID Ancestry Panel for the Eritrean individuals.


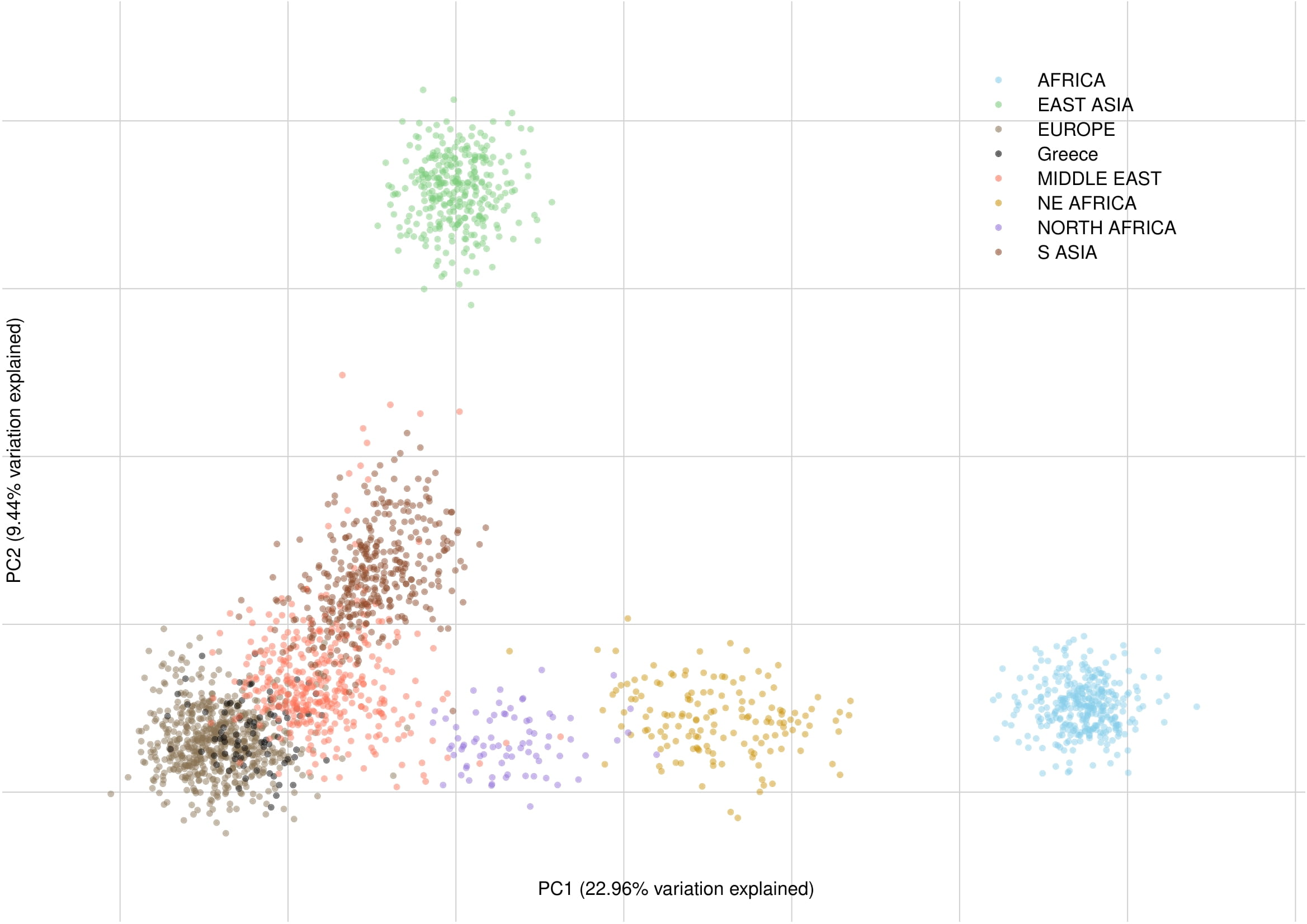


Figure S5: PCA plot of the results obtained with the combined dataset of 233 AIMs included in the EUROFORGEN NAME panel and the Precision ID Ancestry Panel for the Greek individuals.


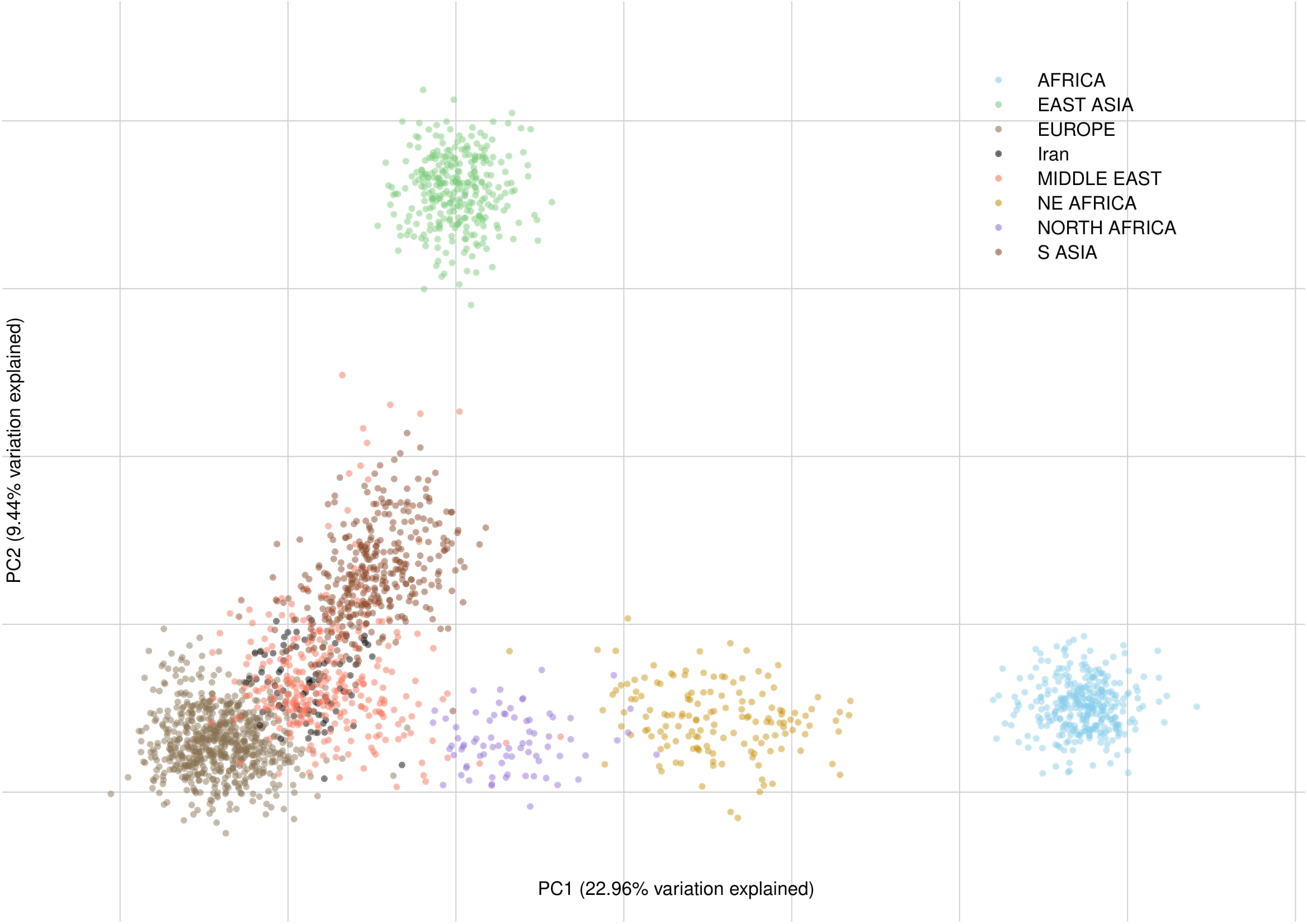


Figure S6: PCA plot of the results obtained with the combined dataset of 233 AIMs included in the EUROFORGEN NAME panel and the Precision ID Ancestry Panel for the Iranian individuals.


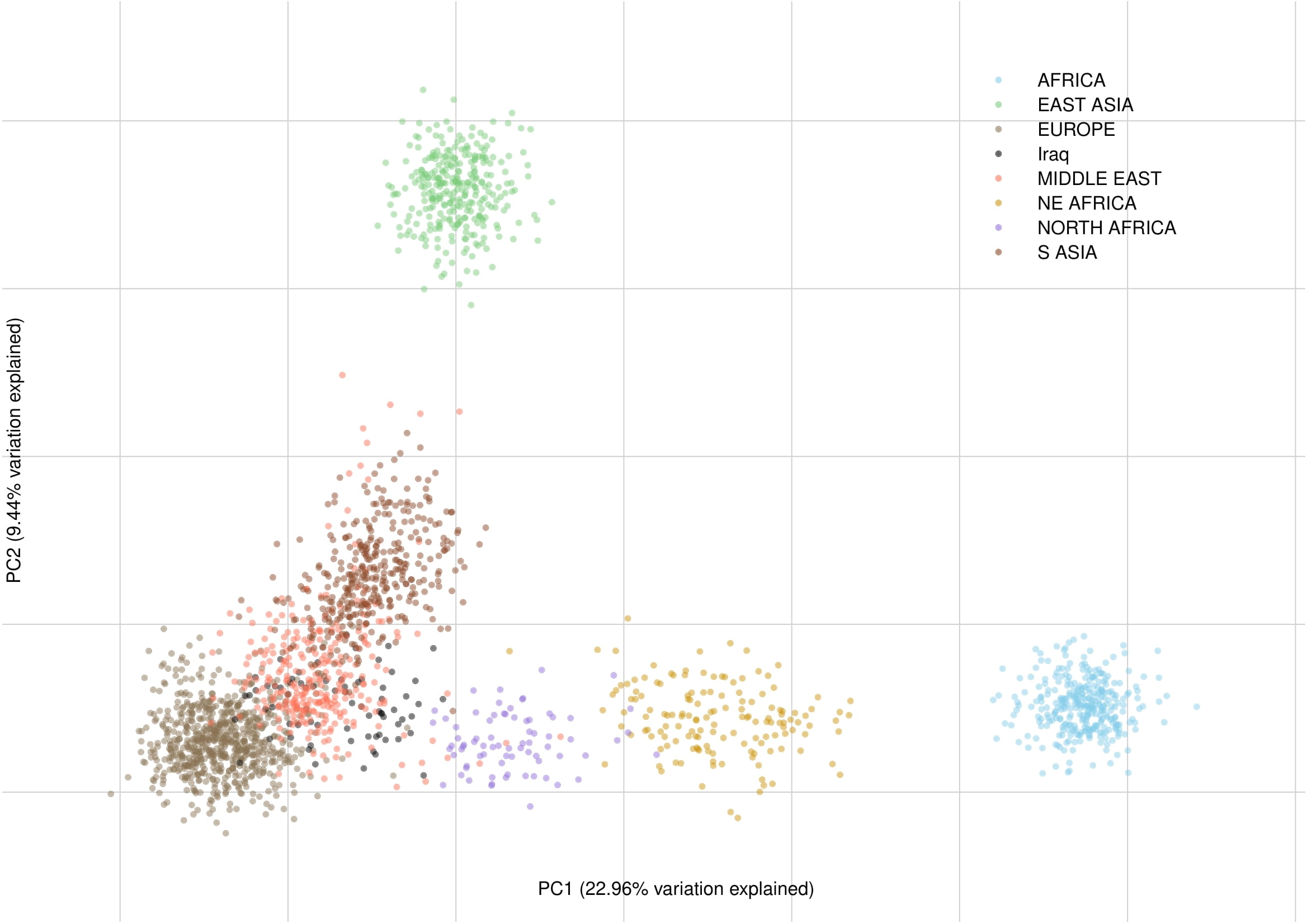


Figure S7: PCA plot of the results obtained with the combined dataset of 233 AIMs included in the EUROFORGEN NAME panel and the Precision ID Ancestry Panel for the Iraqi individuals.


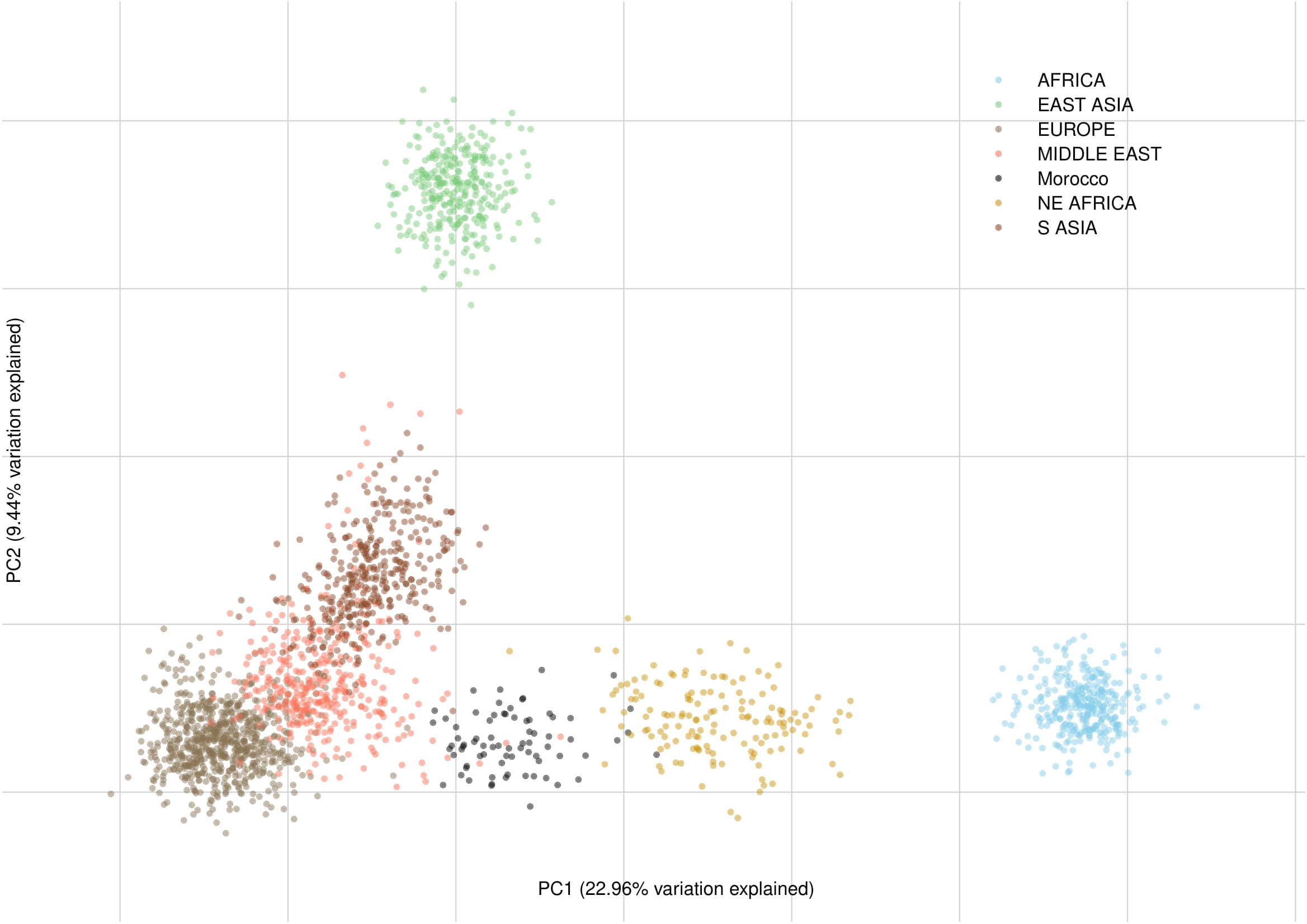


Figure S8: PCA plot of the results obtained with the combined dataset of 233 AIMs included in the EUROFORGEN NAME panel and the Precision ID Ancestry Panel for the Moroccan individuals.


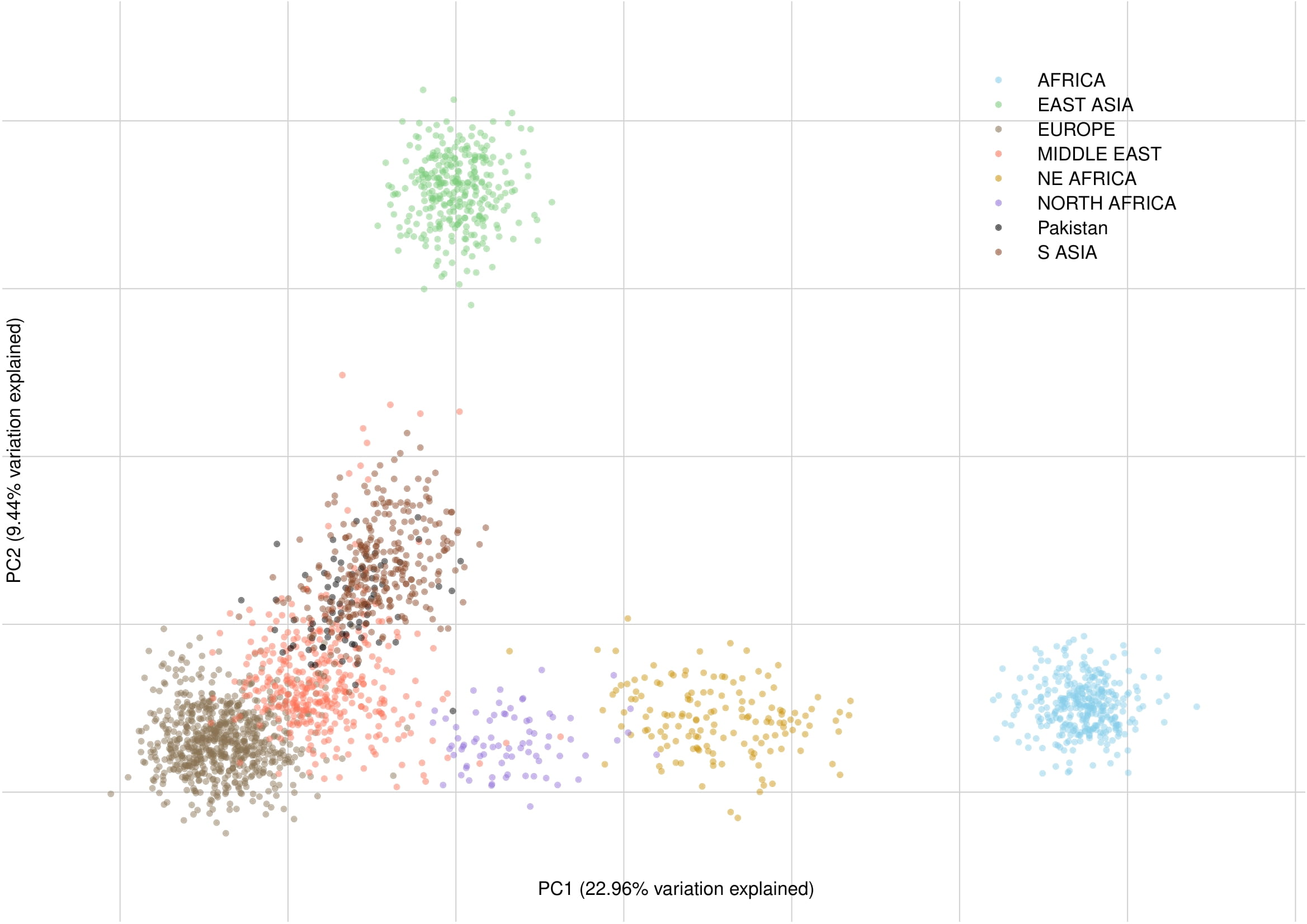


Figure S9: PCA plot of the results obtained with the combined dataset of 233 AIMs included in the EUROFORGEN NAME panel and the Precision ID Ancestry Panel for the Pakistani individuals.


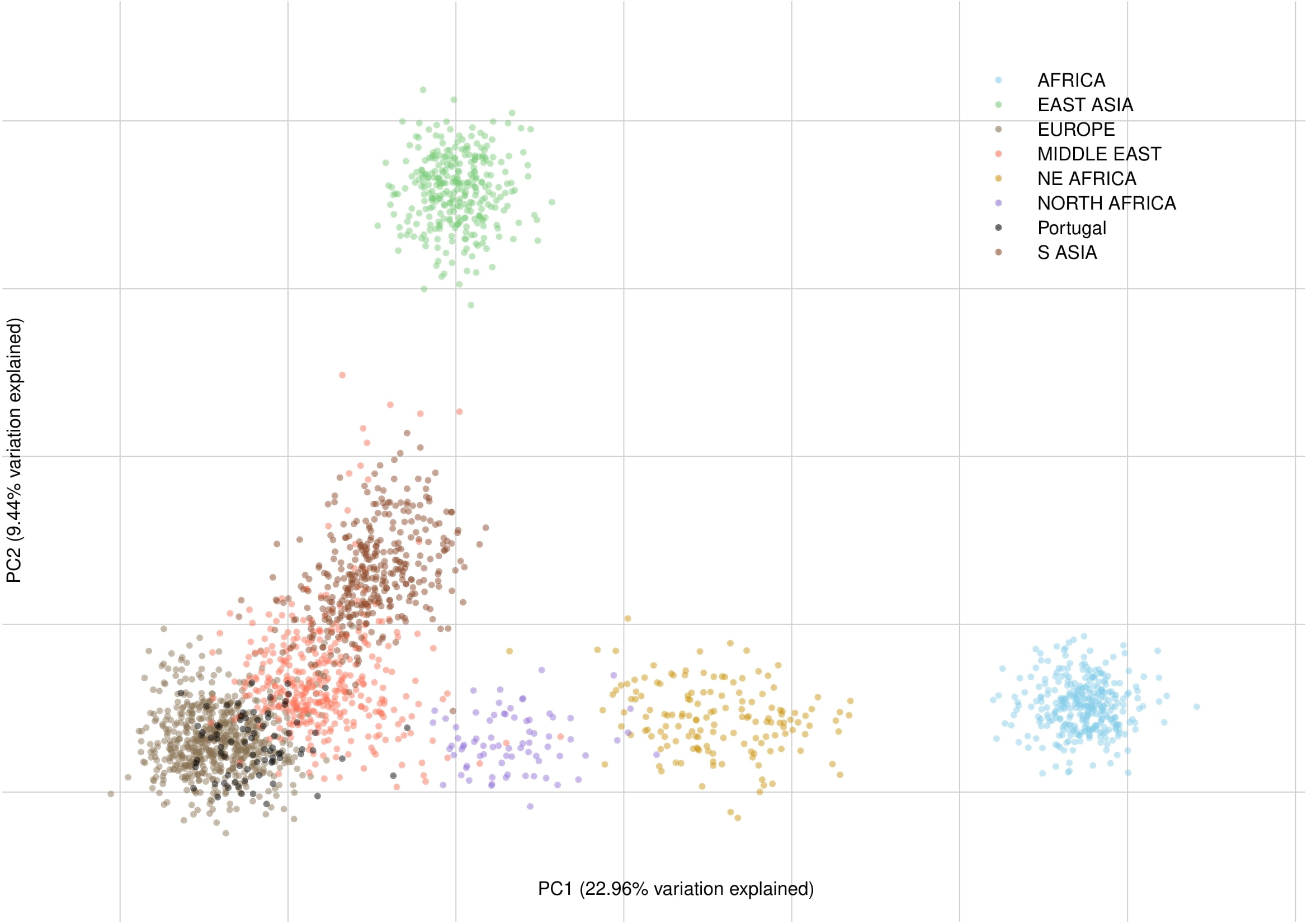


Figure S10: PCA plot of the results obtained with the combined dataset of 233 AIMs included in the EUROFORGEN NAME panel and the Precision ID Ancestry Panel for the Portuguese individuals.


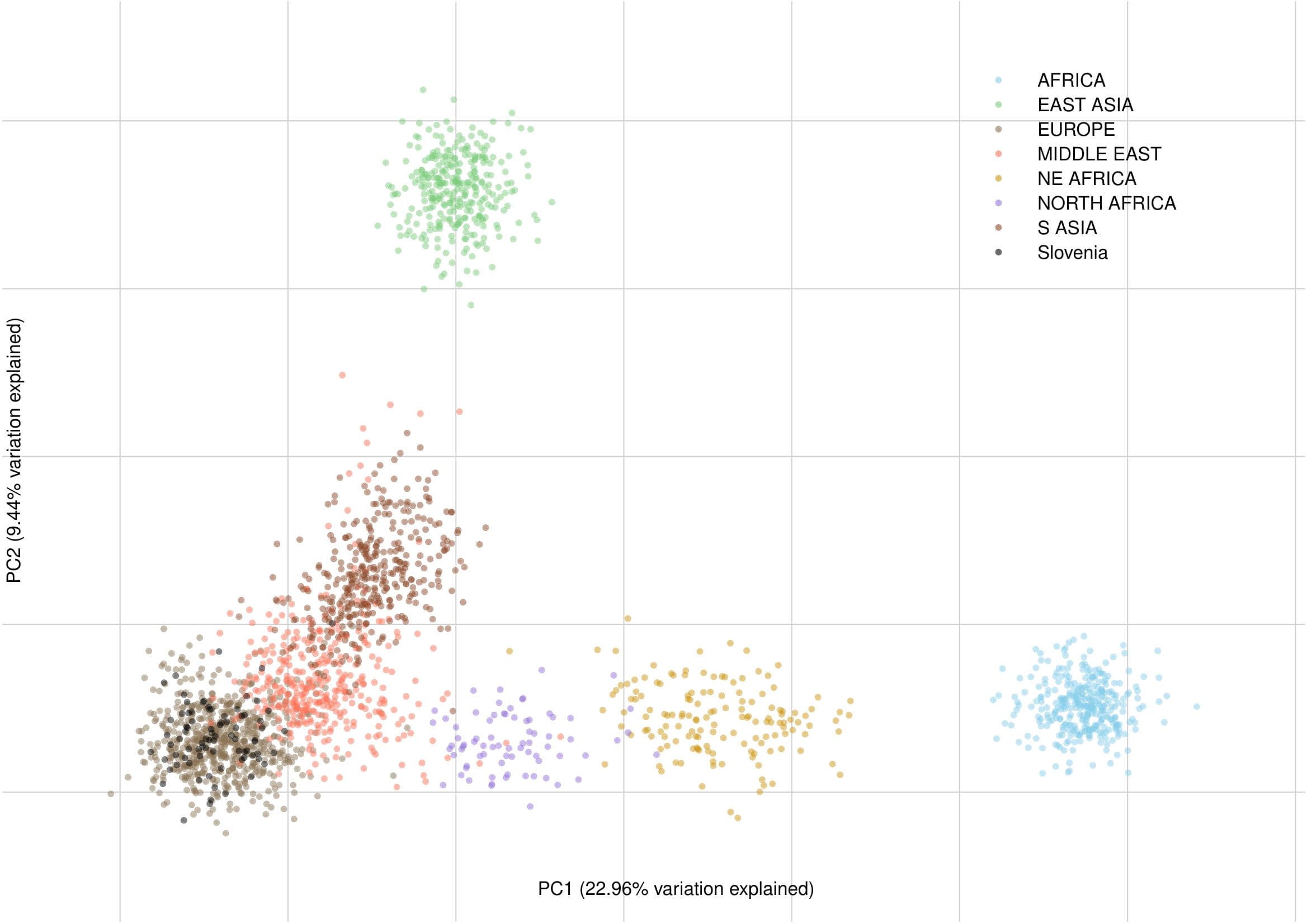


Figure S11: PCA plot of the results obtained with the combined dataset of 233 AIMs included in the EUROFORGEN NAME panel and the Precision ID Ancestry Panel for the Slovenian individuals.


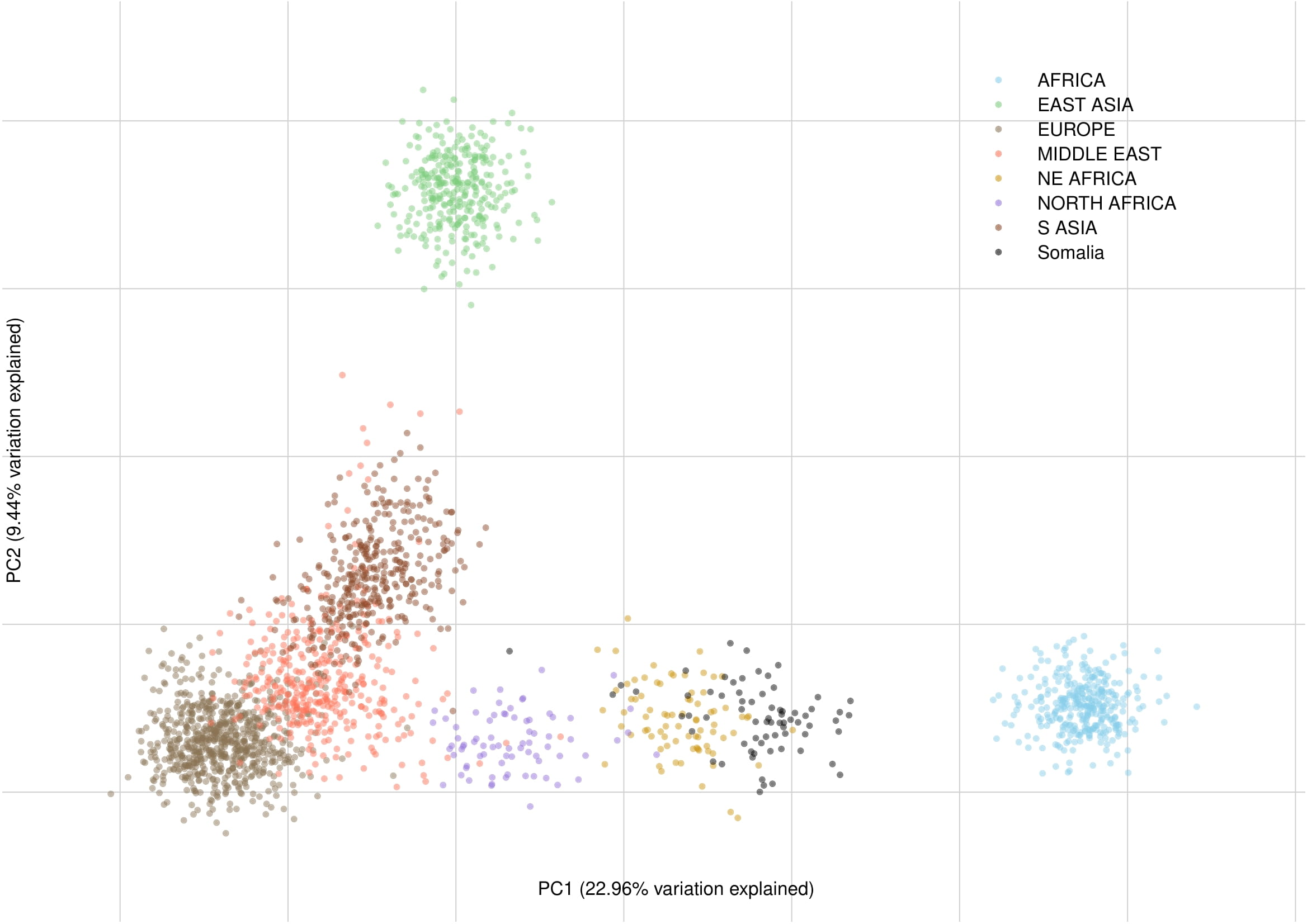


Figure S12: PCA plot of the results obtained with the combined dataset of 233 AIMs included in the EUROFORGEN NAME panel and the Precision ID Ancestry Panel for the Somalian individuals.


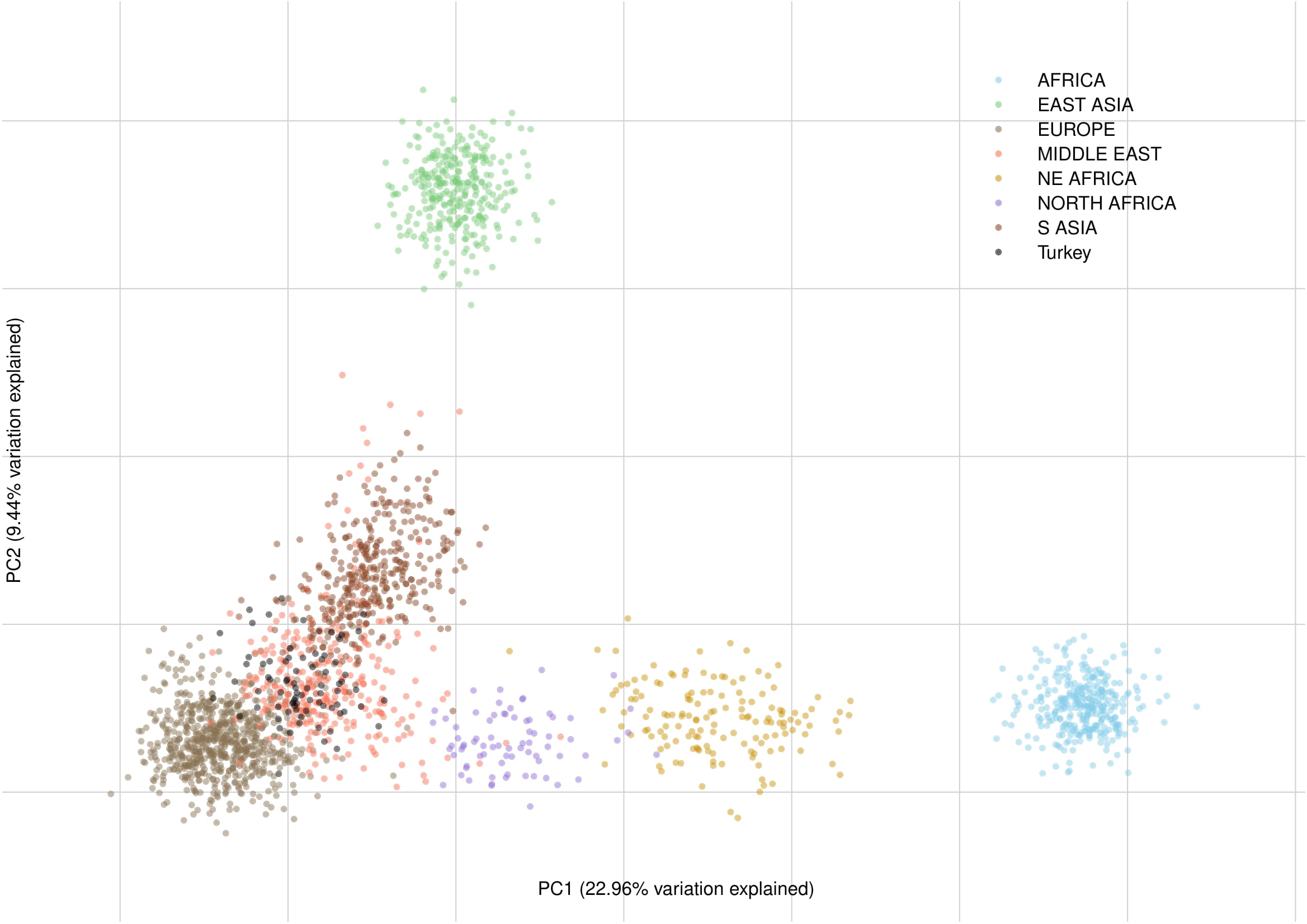


Figure S14: PCA plot of the results obtained with the combined dataset of 233 AIMs included in the EUROFORGEN NAME panel and the Precision ID Ancestry Panel for the Turkish individuals.


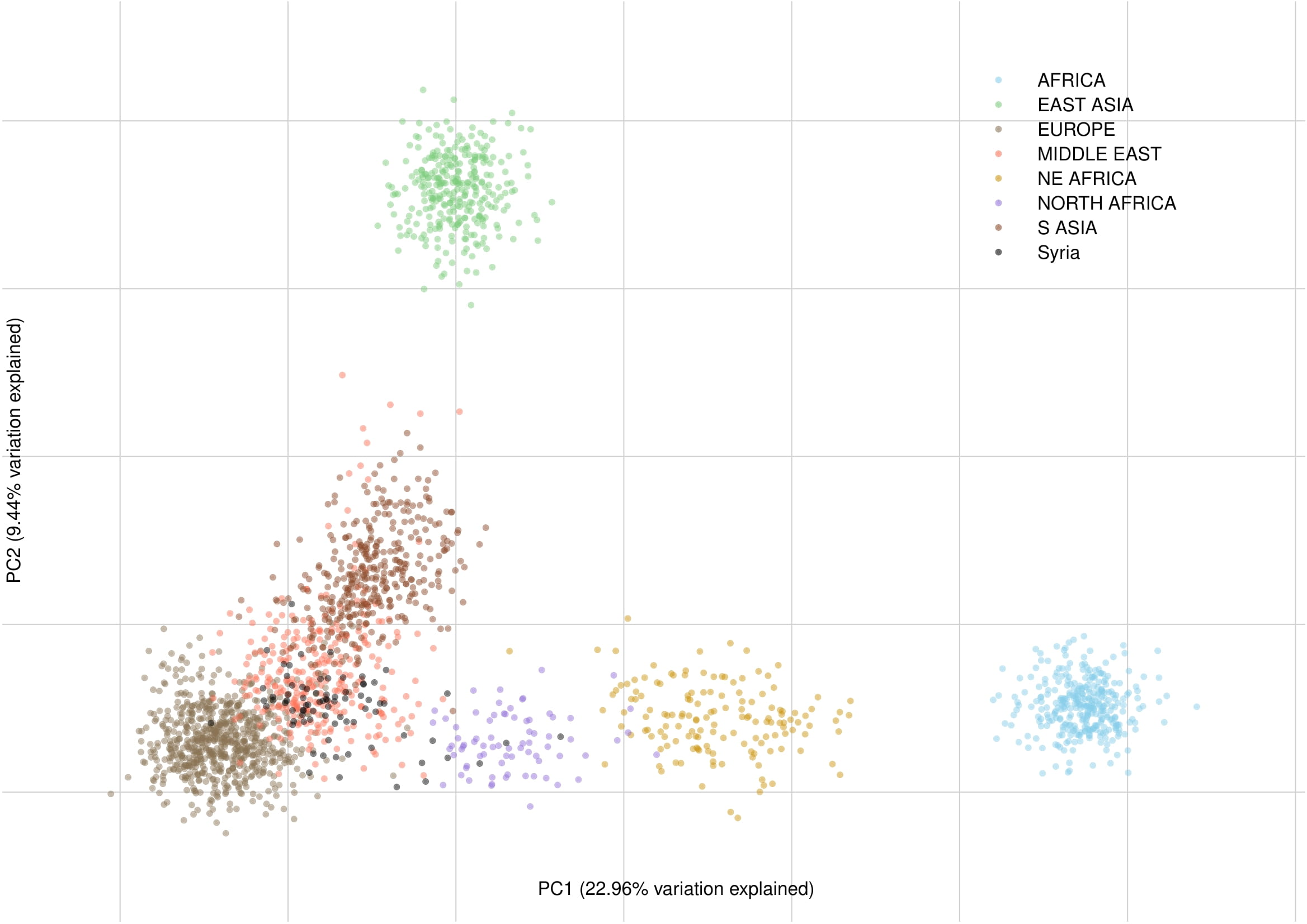


Figure S13: PCA plot of the results obtained with the combined dataset of 233 AIMs included in the EUROFORGEN NAME panel and the Precision ID Ancestry Panel for the Syrian individuals.


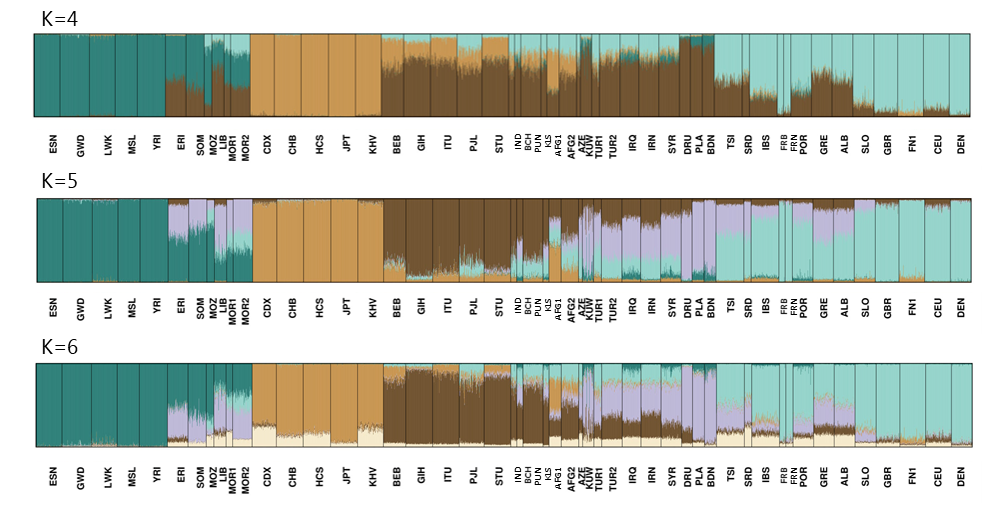


Figure S16: Diagram of the STRUCTURE analysis with runs of K = 4 to 6 of data obtained with the EUROFORGEN NAME markers. The optimal K was 5 based on the analysis performed with Structure Harvester. The reference data are from the 1000 Genomes Project and the HGDP-CEPH panel. Population abbreviations are described in Supplementary Materials Table S2.


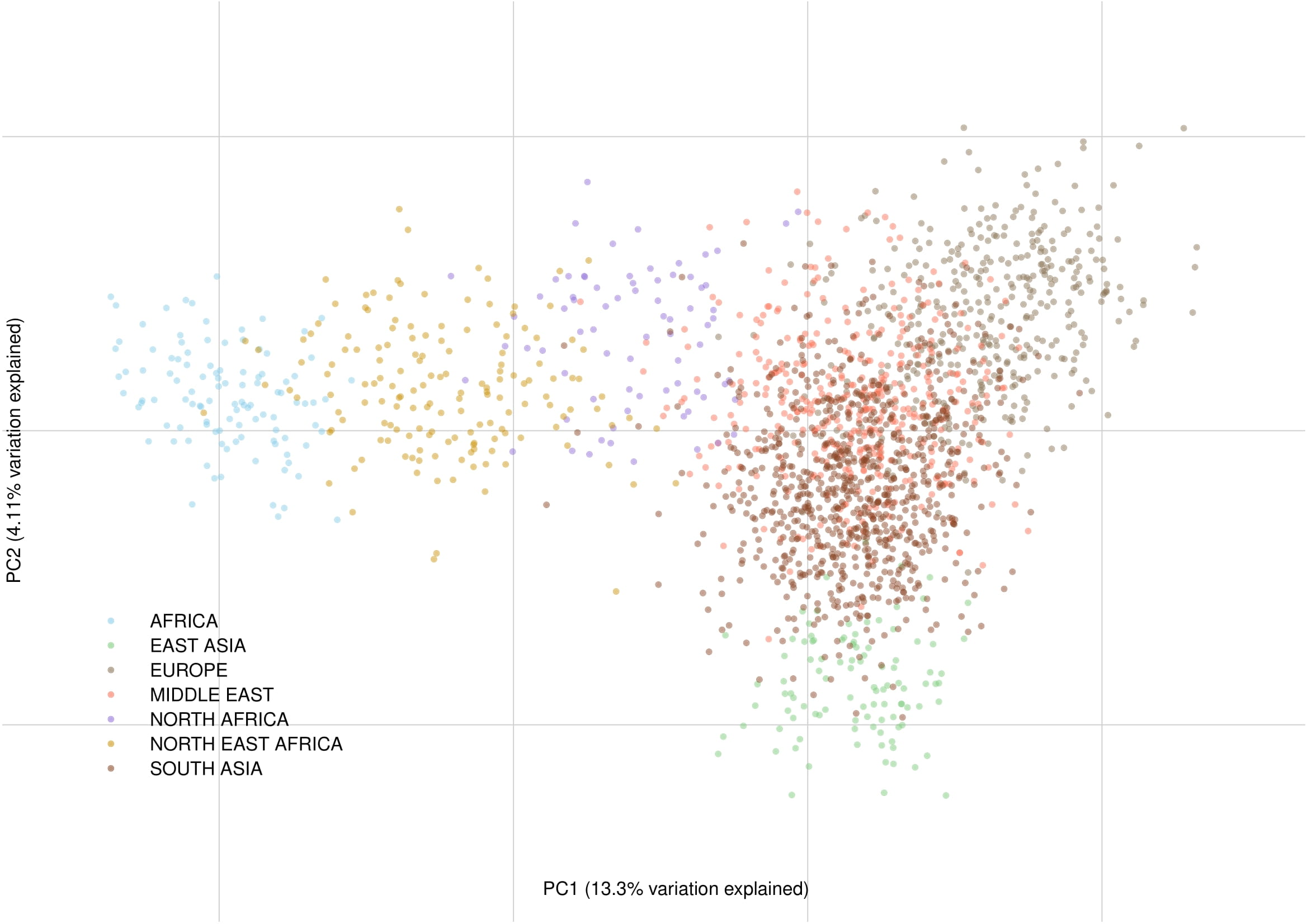


Figure S15: PCA plot based on data of the EUROFORGEN NAME results of 72 AIMs. The reference data were from the 1000 Genomes Project and the HGDP-CEPH panel.
